# Supplementary material for: Shared characteristics of intervention techniques for oral vocabulary and speech comprehensibility in preschool children with co-occurring features of developmental language disorder and a phonological speech sound disorder: protocol for a systematic review with narrative synthesis
Source: BMJ Open. 2023 Jun 1;13(6):e071262. doi: 10.1136/bmjopen-2022-071262 (PMC10255006; doi:10.1136/bmjopen-2022-071262)
Supplement: Supplementary data [file bmjopen-2022-071262supp001.pdf]

SUPPLEMENTARY MATERIALS 1:
SEARCH STRATEGY FOR EACH DATABASE

1.EBSCO *(Medline, APA Psychinfo, CINAHL, Communication Source)*

Paediatric **OR** paediatrics **OR** children **OR** child **OR** infant **OR** infants **OR** schoolchild **OR** schoolchildren **OR** preschool **OR** "early years" **OR** kindergarten *(AB)*
NOT
Teenage **OR** teenagers **OR** adolescent **OR** adolescents *(SU)*

AND

Therapy **OR** intervention **OR** interventions **OR** treatment **OR** treatments **OR** programme **OR** programmes **OR** program **OR** programs **OR** teaching **OR** instruction **OR** approach **OR** approaches **OR** technique **OR** techniques **OR** strategy **OR** strategies **OR** activity **OR** activities **OR** class **OR** classes *(AB)*

AND

"language delay" **OR** "language disorder" **OR** "specific language impairment" **OR** "language impairment" **OR** "language difficulties" **OR** "developmental language disorder" **OR** "late talker" **OR** "speech delay" **OR** "speech disorder" **OR** "speech sound disorder" **OR** "speech intelligibility" **OR** "intelligible speech" **OR** "speech comprehensibility" **OR** "comprehensible speech" *(AB)*
NOT
"sign language" **OR** "mental retardation" **OR** autism **OR** "autistic spectrum disorder" **OR** Asperger **OR** "cleft lip" **OR** "cleft palate" **OR** deaf **OR** "cerebral palsy" **OR** aphonia **OR** geriatrics **OR** "down syndrome" **OR** "cochlear implant" **OR** "autistic spectrum disorder" **OR** "autistic spectrum disorders" **OR** "autism disorder" **OR** "autistic disorder" (*SU)*

Limiters: 2012-current

2.Ovid *(Emcare)*
Paediatric **OR** paediatrics **OR** children **OR** child **OR** infant **OR** infants **OR** schoolchild **OR** schoolchildren **OR** preschool **OR** "early years" **OR** kindergarten *(AB)*
NOT
Teenage **OR** teenagers **OR** adolescent **OR** adolescents *(SH)*

AND

Therapy **OR** intervention **OR** interventions **OR** treatment **OR** treatments **OR** programme **OR** programmes **OR** program **OR** programs **OR** teaching **OR** instruction **OR** approach **OR** approaches **OR** technique **OR** techniques **OR** strategy **OR** strategies **OR** activity **OR** activities **OR** class **OR** classes *(AB)*

AND

“language delay” **OR** “language disorder” **OR** “specific language impairment” **OR** “language impairment” **OR** “language difficulties” **OR** “developmental language disorder” **OR** “late talker” **OR** “speech delay” **OR** “speech disorder” **OR** “speech sound disorder” **OR** “speech intelligibility” **OR** “intelligible speech” **OR** “speech comprehensibility” **OR** “comprehensible speech” *(AB)*
NOT
“sign language” **OR** “mental retardation” **OR** autism **OR** “autistic spectrum disorder” **OR** Asperger **OR** “cleft lip” **OR** “cleft palate” **OR** deaf **OR** “cerebral palsy” **OR** aphonia **OR** geriatrics **OR** “down syndrome” **OR** “cochlear implant” **OR** “autistic spectrum disorder” **OR** “autistic spectrum disorders” **OR** “autism disorder” **OR** “autistic disorder” (*SH*)

Limiters: 2012-current

<u>3.ERIC (descriptor terms)</u>

"speech impairments" **OR** "language impairments" **OR** intelligibility **OR** "expressive language" **AND** "speech language pathology" **OR** "speech therapy" **AND** "young child" **OR** "preschool children" **OR** toddlers **OR** "early childhood education”

Limiters: peer reviewed only
Pre-2012 studies to be removed manually following the search, within Refworks
