# Supplementary material for: Shared characteristics of intervention techniques for oral vocabulary and speech comprehensibility in preschool children with co-occurring features of developmental language disorder and a phonological speech sound disorder: protocol for a systematic review with narrative synthesis
Source: BMJ Open. 2023 Jun 1;13(6):e071262. doi: 10.1136/bmjopen-2022-071262 (PMC10255006; doi:10.1136/bmjopen-2022-071262)
Supplement: Supplementary data [file bmjopen-2022-071262supp002.pdf]

SUPPLEMENTARY MATERIALS 2:
EXCLUSION SCREENING GUIDANCE

Population
Inclusion
80% children aged 2:0 and 5:11; idiopathic phonological speech production difficulties and/or difficulties relating to oral vocabulary; difficulties identified on standardised assessments, parental/and or professional observation reports, and/or pre-intervention baseline probes; idiopathic speech/language needs

Exclusion
Children with typically developing speech/language skills; speech/language difficulties not caused by or associated with a condition with a known impact on communication e.g. autism, deafness, cerebral palsy, cleft lip/palate, dysarthria

Title/abstract level specific: still include if any of the exclusion criteria remains unknown and the inclusion criteria is met or unknown.

Intervention
Inclusion: Any setting, deliverer, mode of delivery

Exclusion: N/A

Comparator
Inclusion: Empirical evaluation of intervention effectiveness from RCTs, experimental and quasi-experimental studies, case studies/within groups designs.

Exclusion: Assessment at a single timepoint pre and post intervention, with no comparator.

Title/abstract level specific: still include if any of the exclusion criteria remains unknown and the inclusion criteria is met or unknown.

Outcome
Inclusion: Measure of oral vocabulary and/or speech comprehensibility. Include intelligibility measures as a proxy for comprehensibility. Include proximal measures of vocabulary development that may arise from syntactic assessments, such as the number of different words (NDW). Outcomes must be evaluated via standardised assessment, probes, and/or observational ratings or scales. Post intervention assessment at any timepoint.

Exclusion: Composite measures where individual results for speech comprehensibility/intelligibility and/or oral vocabulary are not completed in a separate analysis. Purely syntactic measures of change, such as the mean length of utterance in morphemes (MLUm).

Title/abstract level specific: still include if any of the exclusion criteria remains unknown and the inclusion criteria is met or unknown. Still include if primary outcomes do not relate to

vocabulary/speech comprehensibility, but where the inclusion criteria are either met or unknown.
