# Supplementary material for: Shared characteristics of intervention techniques for oral vocabulary and speech comprehensibility in preschool children with co-occurring features of developmental language disorder and a phonological speech sound disorder: protocol for a systematic review with narrative synthesis
Source: BMJ Open. 2023 Jun 1;13(6):e071262. doi: 10.1136/bmjopen-2022-071262 (PMC10255006; doi:10.1136/bmjopen-2022-071262)
Supplement: Supplementary data [file bmjopen-2022-071262supp003.pdf]

SUPPLEMENTARY MATERIALS 3:
DRAFT CONTENT FOR THE DATA EXTRACTION FORM

1.General information/study details

- Date
- Title
- Location (country)
- Language the intervention was in
- Study design
- Comparator
- No. participants (incl. Participants in control/alternative experimental group if relevant)
- Why- goals/aims of the overall intervention
- Specific outcome/s measured of relevance to this review
- Is this a primary outcome of the intervention?
- Do the authors refer to a protocol which was made available before recruitment commenced?
- If a pre-study protocol exists, do the outcomes and results section of the published report align with this protocol?

2.Population characteristics

- Age
- Male/female
- Languages spoken
- Ethnicity
- SES details as reported (e.g. parental education/employment)
- Assessments used to identify DLD/SSD features
- Pre-intervention speech assessment levels
- Pre-intervention language assessment levels (comprehension as well as expressive)
- Phonological SSD sub type- was this specified?
- If phonological SSD sub type was not specified, was it indicated-and if so, how? *(e.g. through the selection of treatment targets, baseline assessment results)*

3.Intervention characteristics

- Setting (e.g. home, nursery/school, clinic)
- Was the intervention modified at any point? If so, how?
- Techniques within the intervention
- Rationale for each technique/the technique within the wider approach
- Mode of delivery of the technique (e.g. fully face to face in clinic; hybrid in clinic with some carryover over home and/or nursery; virtual delivery)
- Was technique delivery implicit (e.g. listening to an adults' model) or explicit (e.g. being asked to repeat)?
- The wider activity/game the technique is part of (e.g. shared book reading, child led play, everyday routines, a combination of these)

- Dose frequency of the individual technique (no. times delivered per session, day, across a week)
- Dose frequency of the intervention as a whole
- Total duration of the technique (the time period between which the technique is used)
- Total duration of the intervention (the time period/duration of the intervention as a whole)
- Additional dosage information as reported
- Deliverer/s of the technique
- If not SLT, how the person was trained to deliver the technique
- Materials used to carry out the technique
- Was intervention fidelity measured? Report on this if so

4.Outcomes

- Summary of exact outcome *(e.g. comprehensibility with parents or with teaching staff? Spontaneous word production in free play or picture naming?)*
- Measure/s used
- Timepoints
- Reported effect
- Direction of effect (benefit vs no benefit/harm)
