# Supplementary material for: Shared characteristics of intervention techniques for oral vocabulary and speech comprehensibility in preschool children with co-occurring features of developmental language disorder and a phonological speech sound disorder: protocol for a systematic review with narrative synthesis
Source: BMJ Open. 2023 Jun 1;13(6):e071262. doi: 10.1136/bmjopen-2022-071262 (PMC10255006; doi:10.1136/bmjopen-2022-071262)
Supplement: Author's manuscript [file bmjopen-2022-071262.draft_revisions.pdf]

BMJ Open

BMJ Open is committed to open peer review. As part of this commitment we make the peer review history of every article we publish publicly available.

When an article is published we post the peer reviewers' comments and the authors' responses online. We also post the versions of the paper that were used during peer review. These are the versions that the peer review comments apply to.

The versions of the paper that follow are the versions that were submitted during the peer review process. They are not the versions of record or the final published versions. They should not be cited or distributed as the published version of this manuscript.

BMJ Open is an open access journal and the full, final, typeset and author-corrected version of record of the manuscript is available on our site with no access controls, subscription charges or pay-per-view fees (http://bmjopen.bmj.com).

If you have any questions on BMJ Open's open peer review process please email info.bmjopen@bmj.com

BMJ Open

The shared characteristics of intervention techniques for oral vocabulary and speech comprehensibility; protocol for a systematic review and narrative synthesis

Journal:	*BMJ Open*
Manuscript ID	bmjopen-2022-071262
Article Type:	Protocol
Date Submitted by the Author:	21-Dec-2022
Complete List of Authors:	Rodgers, Lucy; City University of London, Language and Communication Science; Sussex Community NHS Foundation Trust, Children's Speech and Language Therapy<br>Botting, Nicola ; City University of London, Department of Language and Communication Science<br>Cartwright, Martin; City University of London, Department of Health Services Research and Management<br>Harding, Sam; North Bristol NHS Trust, Bristol Speech and Language Therapy Research Unit<br>Herman, Rosalind; City University of London, Department of Language and Communication Science
Keywords:	Speech pathology < OTOLARYNGOLOGY, Developmental neurology & neurodisability < PAEDIATRICS, Community child health < PAEDIATRICS

SCHOLARONE™
Manuscripts

TITLE PAGE

TITLE:
The shared characteristics of intervention techniques for oral vocabulary and speech comprehensibility; protocol for a systematic review and narrative synthesis

AUTHORS:
Corresponding author: Lucy Rodgers. Department of Language and Communication Science, Myddelton Street Building, City University of London, EC1V 0HB. Lucy.rodgers@city.ac.uk https://orcid.org/0000-0001-6585-7206

Co-author: Prof. Nicola Botting. Department of Language and Communication Science, City University, London, UK.

Co-author: Dr Martin Cartwright. Department of Health Services Research and Management, City University, London, UK.

Co-author: Dr Sam Harding. Bristol Speech and Language Therapy Research Unit, North Bristol NHS Trust, Bristol, UK. https://orcid.org/0000-0002-5870-2094

Co-author: Prof. Rosalind Herman. Department of Language and Communication Science, City University, London, UK. http://orcid.org/0000-0001-5732-9999

Word count (excluding title page/abstract/references): 3,725

Key words: Language Development Disorders; Speech Sound Disorder; Child Language; Speech-Language Pathology

For peer review only

ABSTRACT

Introduction:

Evidence suggests that over a third of young children with a speech sound disorder (SSD) or developmental language disorder (DLD) have co-occurring features of both. A co-occurring SSD and DLD profile is associated with negative long-term outcomes relating to communication, literacy and emotional wellbeing. However, the best treatment approach for young children with this profile is not understood. The aim of the proposed review is to identify intervention techniques for both SSD and DLD, along with their shared characteristics. The findings will then be analysed in the context of relevant theory. This will inform the content for a new or adapted intervention for these children.

Methods and analysis:

This search will build on a previous systematic review by Roulstone et al. (2015) but with a specific focus on oral vocabulary (DLD outcome) and speech comprehensibility (SSD outcome). These outcomes were identified by parents and Speech and Language Therapists within the pre-study stakeholder engagement work. The following databases will be searched for articles from January 2012 onwards: Ovid EMCare, Medline Complete, CINAHL, APA PsychINFO, Communication Source and ERIC. Two reviewers will independently perform the title/abstract screening and the full text screening with the exclusion criteria document being revised in an iterative process. Data will be extracted regarding key participant and intervention criteria, including technique dosage and delivery details. This information will then be pooled into a structured narrative synthesis.

Registration details: Prospero registration number CRD42022373931. In the event of protocol amendments, the date of each amendment will be accompanied by a description of the change and the rationale.

Ethics and dissemination: Ethical approval is not needed for a systematic review protocol. Dissemination of findings will be through peer-reviewed publications, social media, and project steering group networks.

Strengths and limitations of this study

- **Strength:** This protocol follows the referred Reporting Items for Systematic Review and Meta-Analysis Protocols (PRISMA-P) guidelines.
- **Strength:** The proposed systematic review addresses a critical research gap by systematically identifying intervention techniques of relevance to children with co-occurring SSD/DLD, and reviewing their alignment to current theory. This has long term implications regarding the development of future interventions for this vulnerable group.
- **Limitation:** Electronic databases in languages other than English will not be searched. This may cause language bias.
- **Limitation:** Meta-bias(es) within the literature cannot be fully controlled. This may exacerbate reporting and publication bias.

For peer review only

- **Limitation**: The level of detail within intervention reporting, as per the TIDieR guidelines, has the potential to vary amongst studies.

INTRODUCTION

Co-occurring Speech Sound Disorder (SSD) and Developmental Language disorder (DLD)

An estimated 7.58% of 4-year olds present with features of a Developmental Language Disorder (DLD) (1). DLD is characterised by idiopathic difficulties in using and understanding spoken language (2). DLD may co-occur with Speech Sound Disorder (SSD); that is, difficulties in producing speech sounds (3). An estimated 3.4% of 4- year olds have SSD (4). Thirty six percent of 4-year olds with idiopathic SSD also have oral (i.e. spoken) language features of DLD (4). This high rate of co-occurrence is in keeping with historical research in the area (5), as well as study data from clinical caseloads (6). Co-occurring features of SSD/DLD in early childhood are associated with negative long-term outcomes relating to literacy (7,8) and communication (8,9), with downstream consequences for quality of life (10,11) and emotional wellbeing (12). Therefore, it is essential that young children with this co-occurring profile can access early, targeted intervention (13). Presently, little is known about the best practice and most effective treatments for the targeting of speech and language concurrently. To advance understanding in this field, this protocol outlines the systematic review process for the bringing together of evidence for paediatric SSD and DLD interventions.

The overlap between SSD and DLD is underpinned by shared linguistic deficits of both disorders (14,15). This is particularly evident when the DLD co-occurs with a phonological SSD, whereby the child has difficulties with manipulating the different sound contrasts (phonemes) which are needed to form words (16). Within the early years of life, complex and bi-directional relationships between the development of individual sounds (phonology) and words (the lexicon) have been identified (17,18). For example, the first words of young children primarily consist of the speech sounds already established within their emerging phonological inventory (18). This relationship between phonology and the lexicon may have implications for intervention with children with co-occurring features of SSD/DLD. For example, growth in vocabulary and/or the strengthening of phonological representations has the potential to impact speech and vocabulary development concurrently through a process known as ‘lexical restructuring’ (19). A further psycholinguistic theory of potential relevance is the speech processing model (20), which suggests that individual children with SSD/DLD may have difficulties at one or more levels of speech processing, rather than just with phonological representations alone. Such theories are important within interventions for co-occurring SSD/DLD, as underlying linguistic theory has the potential to guide the content and delivery of interventions for co-occurring SSD/DLD, so that language and speech may be targeted concurrently (21).

Current Interventions for co-occurring SSD/DLD

‘Child Talk’ (22) was a large National Institute of Health Research (NIHR) funded mixed methods programme of work, including a systematic review. This involved investigating the

For peer review only

use of early years' speech and language therapy interventions. The findings led to the specification of a) a typology of early years' Speech and Language Therapy (SLT) intervention, and b) key intervention techniques for each typology theme. A technique can be described as "the specific teaching behaviours/actions thought to effect change" (23). For the purpose of this article and reflecting the 'Child Talk' findings (22) the term technique may include the use of strategies such as recasting, or specific therapy activities such as minimal pairs (24).

Several interventions for children with features of DLD *or* SSD have been evaluated and found to be effective (25-29). However, the findings of 'Child talk' (22) highlighted that for children with co-occurring SSD/DLD features, clinicians often adapt existing interventions by selecting and combining techniques from different interventions. This enables them to use their knowledge and experience to provide the best treatment that they can (22,30).

Although it is important to tailor intervention to meet the needs of individual children, our knowledge of what techniques work best and why, in relation to co-occurring features of SSD/DLD, is limited. Despite the shared linguistic underpinnings of both disorders, there is a paucity of theoretically informed intervention research which can be easily translated into everyday clinical practice (24). Due to this paucity of evidence, the associated negative impact of the co-occurring condition on long term outcomes, and the high level of presentation within clinical caseloads, there is an urgent need to establish effective interventions for this clinical group.

Broader context: an intervention development study

The proposed review updates the systematic review findings from 'Child Talk' (22) whilst refining the focus to techniques within SSD/DLD interventions. We will be extracting descriptions of techniques from articles for SSD or DLD. These techniques will then be analysed in relation to shared characteristics and underpinning theory. The synthesis will then be used to inform the content of a new intervention which is being developed for young children with co-occurring features of SSD/DLD.

Based on the dose form framework (23), shared characteristics for SSD/DLD intervention techniques may include similarities in:

1) *who* delivers the technique; for example, is it the parent, clinician, or both?
2) *where* the technique is delivered; for example, at home, nursery, clinic or a combination of these?
3) the nature of *technique delivery*; for example, is it during an adult led structured game, child led play, everyday routines, or a combination of these?

Underpinning theory may relate to:

1)The lexical restructuring hypothesis (19)
2) Psycholinguistic models of speech and language development; such as the speech processing model (20)

For peer review only - http://bmjopen.bmj.com/site/about/guidelines.xhtml

3)The neural basis for speech and language development; for example, the role of meaningful interactions within language learning (31)

Defining outcomes and wider relevance: Pre-study Patient and Public Involvement and Engagement (PPIE)

According to the James Lind Alliance, knowing how to best select communication strategies according to a child's individual profile is the 2nd most important recommendation for research (32). This is strongly in keeping with the aims of this review, and highlights the broader relevance of this work.

Both SSD and DLD are heterogenous disorders (2,33), and therefore have a range of associated outcomes. For our wider intervention development study, outcomes were prioritised by clinicians and parents of children with SSD/DLD within pre-study PPIE work (34,35). They identified the outcomes of increasing 1) oral vocabulary *(DLD outcome)*, and 2) speech comprehensibility *(SSD outcome)*. Consequently, this review will focus exclusively on techniques that directly target oral vocabulary and speech comprehensibility.

Registration: In accordance with the guidelines, our systematic review protocol has been registered with the International Register of Systematic Reviews (PROSPERO) on 16/12/2022 (registration number CRD42022373931).

Objectives

The overarching aim of the review is to bring together intervention techniques from DLD and SSD interventions. The objectives within this are to:

1. Identify the shared core characteristics of the techniques; this includes the deliverer, place of delivery, format of delivery and nature of delivery *(e.g. child or adult led)*

2. Compare and synthesise the shared core characteristics of the techniques in relation to underlying theory

3. Establish the best available evidence for interventions that incorporate these core characteristics of the intervention techniques

Research questions:

1)What are the shared core characteristics of intervention techniques in preschool interventions targeting speech comprehensibility and/or oral vocabulary?

2)How do these shared core characteristics relate to underlying theory?

3)What evidence is there for the effect of interventions that incorporate these core characteristics of intervention techniques?

METHODS AND ANALYSIS

Eligibility criteria

The eligibility criteria stated below are in line with the criteria from the original 'Child Talk' systematic review (22) with amendments according to the objectives of the current review. Most importantly, this review will focus specifically on the 'expressive language' and 'speech' themes generated from their initial typology of early years' Speech and Language Therapy (SLT) interventions, as these themes encompass the two outcomes for which we are seeking to identify techniques.

Study designs

Included studies must report on an empirical evaluation of the effectiveness of an intervention. To ensure we identify all relevant literature, a range of study designs will be included. These include randomised control trials (RCTs), experimental and quasi-experimental studies, within subjects designs (e.g. pre-post studies) and case studies (which may include multiple baseline or other systematic manipulation of the intervention). Studies which report on single timepoint (e.g. cross-sectional studies) will be excluded.

Population

To capture the age group most typically seen within clinical services, 80% of children within included studies must have been aged between 2:0 and 5:11 years. They must have presented with phonological speech production difficulties and/or difficulties relating to oral vocabulary, as identified by standardised assessments such as the Preschool Language Scale (36), parental and/or professional observation reports such as the intelligibility in context scale (37) and/or probes. Probes may also be used to assess progress through the repeated measurement of the dependent variable before, during and after the intervention. As already observed in the literature, common probes within speech and language therapy interventions may include a selection of words containing the child's targeted speech sound/s or vocabulary (28,38). Included papers must state that the participants' needs had no obvious cause, i.e., excluding children with neurodevelopmental differences that have a known association with speech and/or language development, such as Autism or Cerebral Palsy.

Intervention

We will include studies reporting on interventions delivered in any setting (e.g., home based, clinic) or format (e.g., face to face, online). The deliverer may be a speech and language therapist, speech and language therapy assistant, or equivalent professional, and the intervention may involve professionals training up others (e.g., parents) to deliver some or all of the intervention.

Comparator

Comparators for included studies may be a control without an intervention (including multiple baseline and within subjects designs) or an alternative experimental group (i.e., intervention comparison).

Outcomes

For peer review only

Included papers must measure the effectiveness of the intervention on a) oral vocabulary, and/or b) speech comprehensibility. These outcomes must be evaluated via standardised assessment, probes, and/or observational ratings or scales.

If composite speech and language assessments are used, studies must report on the separate sub test results for oral vocabulary and/or speech comprehensibility to be included.

Studies with only syntactic measures of language change will be excluded; this includes mean length of utterance in morphemes (MLUm). However, they will be included if a proximal measure of vocabulary change is used alongside syntactic measures, such as the number of different words (NDW).

Historically, there has been uncertainty over when to use the terms speech comprehensibility and speech intelligibility (39), which are overlapping but different constructs. This has led to them being used inter-changeably within the literature. Both terms relate to functional human communication (40). However, they differ in that intelligibility refers to the acoustic-phonetic decoding of utterances, whereas comprehensibility refers to the reconstruction of the meaning of the message (40). Therefore, we use the term 'comprehensibility', because our focus is on a child's ability to produce speech which is understandable to others within meaningful everyday environments. However, we will still include studies with an outcome of improved speech intelligibility as a proxy for comprehensibility, due to this shared focus on functional human communication.

Information sources

The search will be conducted in Ovid EMCare, Medline Complete, CINAHL, APA PsychINFO, ERIC and Communication Source. These sources have been selected as they encompass the fields of health (medical, nursing and allied health professions), speech and language therapy, education and psychology and have been successfully utilised in previous reviews in the field (24,41). To support with literature saturation, supplementary search methods will be employed; this includes screening the reference lists from prominent reviews in the field post 2012 (41,42,43). We have selected reviews from 2012 onwards due to the original search going up to this date (22). Reference lists from included papers within the current search will also be screened for potential study eligibility. Forward citation searches in Web of Science (using the core collection) will also be carried out, with additional searches in Scopus if the titles are not available in Web of Science.

Due to resource constraints, articles written in languages other than English will be excluded. However, articles written in English where the participants speak languages other than English will be included. Additionally, grey literature searching will be confined to the inclusion of theses/dissertations, via the databases stated above. Thesis/dissertations have been selected as although the original review (22) included a range of grey literature, thesis/dissertations were the only grey literature sources which contributed studies within the final included papers. In keeping with the original review, thesis/dissertations will only be included when a corresponding journal article cannot be found for the study.

Search strategy

Together with support from a specialist librarian, we will conduct an update of the original 'Child Talk' systematic review (22), searching articles from January 2012 to the present day. One of the researchers (SH) undertaking the current search also led on the original review. Relevant studies from the original 'speech' and 'expressive language' typology themes within the original 'Child Talk' review have already been located by reviewing the recorded outcomes for each study as stated on the original data extraction spreadsheet. Out of 41 papers from the 'speech' theme, two were found to address the outcome of comprehensibility. From the 30 papers within the 'expressive language' theme, 12 were found to include oral vocabulary as an outcome. These 14 papers will be further screened at stage 2 of the screening process (full text stage, outlined below).

The original review search strategy (22) has been updated for the current review, accounting for advances in terminology, e.g., consensus on the term 'Developmental Language Disorder' (2). The original 'Child Talk' search encompassed a broader range of speech and language outcomes, therefore the search terms for the current review have been adjusted to focus on our two specific outcomes of interest; oral vocabulary and speech comprehensibility. The updated search strategy was initially reviewed by two independent post-doctoral researchers in the field and adjusted as needed, for example, adding in the term 'specific language impairment', which may be relevant to older papers in the search. For the revised search strategy draft, please see supplementary material 1.

Study records, Selection and data collection process

Search results will initially be imported into RefWorks, where duplicates will be removed by the first author (LR). The remaining articles will then be uploaded to the Covidence systematic review management database.

Initially, the first author (LR) will trial the exclusion guidance criteria document on 30 papers. For the initial draft of this exclusion criteria guidance document, please see supplementary material 2. The 30 papers will be randomly selected using a random number generator. These 30 papers will then be reviewed by a second reviewer. The reviewers will then meet to discuss discrepancies and make amendments to the exclusion guidance document if needed.

The screening and data extraction will be carried out as follows:

1) *Title/abstract screening*
   The full set of titles/abstracts will be screened by the first author (LR). If uncertainty arises about how to apply to eligibility criteria to a specific paper, these articles will be discussed with a member of the review team (who is not involved in the formal screening process). This may then lead to further revisions to the exclusion criteria document. Following this, a second independent reviewer (SH) will independently screen the titles/abstracts. Any disagreements, and how these may relate to the exclusion guidance document, will be discussed in consensus meetings. Any disputed

For peer review only - http://bmjopen.bmj.com/site/about/guidelines.xhtml

articles will then be re-screened should alterations have been made to the exclusion criteria document. If disagreement is not caused by confusion over the exclusion criteria document, and consensus cannot be reached through discussion, a third reviewer will be consulted.

2) *Full paper screening*
At the full text screening stage, two reviewers (LR, SH) will independently appraise all of the remaining articles for inclusion, following the iterative process as outlined for stage 1.

To enable transparency of the reliability of screening at stages 1 and 2, Cohen's K for these stages will be reported in the final paper.

3) *Risk of bias/internal validity*
Retained studies will then undergo assessment of internal validity by two independent reviewers (LR, SH). The reviewers will have regular consensus meetings, after independently assessing up to 4 papers at a time, to resolve potential conflicts. If disagreements persist, a third reviewer will be involved. Disagreements that arise (including those that have been resolved) will be recorded and reported in the final paper.

For the PEDro-P (44), papers with a rating of six and over will be retained for data extraction. This aligns with the original review (22). On the Risk of Bias in N-of-1 Trials (RoBiNT) scale, included studies will be rated as *fair* or above (45).

4) *Data extraction*
The first author (LR) will extract data from the first 25% of studies. These will be randomly selected using random number generation. A second extractor (SH) will then independently extract data from the same studies. The two extractors will then meet to discuss potential discrepancies, and to update the data extraction form if needed. Following this, the first author (LR) will extract the remainder of the data.

Data items

Data will be sought regarding general study information (e.g., date; study type; location; participant numbers), population characteristics (e.g., male/female; age; speech/language profile), intervention techniques (e.g., dosage; underpinning theory and justification given by the authors) and reported impact on the outcome of interest. Data on reported participant SES background will also be obtained, due to this being a known risk factor within developmental speech and language disorders (46). We will also collate information on the number of languages spoken by the participants, as well as reported ethnicities, with this being a potential factor for the external validity of findings (i.e., relevance to 'everyday' clinical practice).

The Template for Intervention Description and Replication (TIDieR) will be used as a framework to guide the extraction process (47), combined with the speech and language

For peer review only

therapy specific 'Dose form Framework and Definitions', based on the work of Warren and colleagues (48); this has been applied in other reviews specific to paediatric speech and language therapy intervention (23). Details of techniques will be extracted regarding intervention contexts (e.g. the overarching activity the technique is presented in), method of instruction (e.g. who delivers the technique, where and when), and technique dosage (dose frequency and dose duration). For the initial draft of the data extraction form, please see supplementary materials 3.

Outcomes and prioritisation

The two outcomes (oral vocabulary, speech comprehensibility) are of equal interest within this review, regardless of whether they are primary or secondary outcomes within the included studies.

Risk of bias in individual studies

Individual studies will be assessed for internal validity. To encompass the range of study designs included within this review, we will use the PEDro-P (44). Specifically, for single case experimental designs, the RoBiNT scale will be used (45).

Data synthesis

Quantitative data
Overarching details for each included study, including the individual internal validity ratings, will be given in the first table. Two summary graphs will also be presented to convey the percentage of overall ratings from the PEDro-P and RoBiNT Scales. The frequency of techniques within the included papers will be presented either numerically within a table, or within a graph or chart if this deemed more suited to the data collected. We will be guided by the synthesis without meta-analysis (SWiM) in systematic review guidelines (49) and will report on the direction of effect of the interventions, using vote counting with a sign test if appropriate.

Qualitative data
A description of the identified techniques will be presented in a table, including details regarding how they were operationalised, based on TIDieR (48) and the dose form framework (23).

The narrative synthesis will include sections on:
1)Similarities and differences (including shared core characteristics) between techniques used for the different outcomes
2)Patterns of technique dosage and delivery across the interventions
3)How the similarities and differences (including shared core characteristics) in techniques relate to underlying theory
4)The effectiveness of interventions which contain these techniques/shared core characteristics of techniques

PPIE in data collection, analysis and dissemination

For peer review only

In keeping with the integral role of PPIE throughout, a newly formed project PPIE steering group will provide input at key points in the review process. This is a diverse group consisting of parents, speech and language therapists, a person with DLD, a specialist early years educator, a bi/multi-lingual educational family support worker and a clinical equality, diversity and inclusion (EDI) expert. During the review, they will be involved with:

1) Reviewing the content for the data extraction form (supplementary document 3), prior to the data extraction phase

2) Identifying what data has the most relevance in the 'real world', with these potentially informing recommendations within the final paper

3) Defining and agreeing key messages to take from the review, and dissemination through the steering group networks.

Steering group input will be recorded and reported in the final article, in accordance with the GRIPP 2 reporting criteria short form (50).

Meta-bias (es)

It is important to acknowledge that meta-bias, including reporting and publication bias, is present within all aspects of health research. Although it is not possible to completely control for such bias, we will:

1) Establish if the protocol for each study was published before recruitment for participants commenced (where possible)

2) Compare the outcomes and results sections of the published report when a protocol is available (for when considering selective reporting bias)

3) Assess potential publication bias through the inclusion of prioritised grey literature (thesis/dissertations)

Confidence in Cumulative evidence

Confidence within the evidence as a whole will be based on the summary of the internal validity, as presented in the two summary tables (see data synthesis section). We will also acknowledge and discuss key factors relating to meta bias, and how the review findings should be interpreted based on this.

SUMMARY

This systematic review will build on the prior review (22) with a specific focus on the outcomes of 1) oral vocabulary, and 2) speech comprehensibility. Our review will identify intervention techniques relating to these two outcomes of interest, which can then be analysed in the context of similarities, differences and underlying theory. Findings will then be taken forward for further consideration within later phases of the project, in order to

embed both research evidence and potential theories of relevance into the development of a new intervention. Additionally, clinical recommendations for current practice may be made should the nature of the evidence indicate appropriateness for this.

Ethics and dissemination:
As a systematic review this study does not warrant ethics board approval. Findings will be disseminated though peer reviewed publications, social media, and project steering group networks.

Authors' contributions
LR led on this work and independently developed an initial draft of the manuscript (and appended documents). RH, NB, MC and SH suggested amendments after reviewing this and 3 subsequent re-drafts by LR. LR completed the fourth and final version of the manuscript, which was reviewed and agreed by all of the authors.

The authors would also like to acknowledge and thank Dr Pauline Frizelle (University College Cork) and Dr Helen Stringer (Newcastle University) for providing expert independent reviews of the initial terms for the search strategy.

Competing interests' statement
There are no competing interests to declare.

Funding
This research was funded in whole, or in part, by the Wellcome Trust (Grant number 223500/Z/21/Z). For the purpose of open access, the author has applied a CC BY public copyright licence to any Author Accepted manuscript version arising from this submission.

For peer review only

FULL REFERENCES

1) Norbury, C.F., Gooch, D., Wray, C., Baird, G., Charman, T., Simonoff, E., Vamvakas, G. and Pickles, A., 2016. The impact of nonverbal ability on prevalence and clinical presentation of language disorder: Evidence from a population study. https://doi.org/10.1111/jcpp.12573
2) Bishop, D.V., Snowling, M.J., Thompson, P.A., Greenhalgh, T., Catalise-2 Consortium, Adams, C., Archibald, L., Baird, G., Bauer, A., Bellair, J. and Boyle, C., 2017. Phase 2 of CATALISE: A multinational and multidisciplinary Delphi consensus study of problems with language development: Terminology. https://doi.org/10.1111/jcpp.12721
3) Dodd, B., Reilly, S., Ttofari Eecen, K. and Morgan, A.T., 2018. Articulation or phonology? Evidence from longitudinal error data. https://doi.org/10.1080/02699206.2018.1488994
4) Eadie, P., Morgan, A., Ukoumunne, O.C., Ttofari Eecen, K., Wake, M. and Reilly, S., 2015. Speech sound disorder at 4 years: Prevalence, comorbidities, and predictors in a community cohort of children. https://doi.org/10.1111/dmcn.12635
5) Shriberg, L.D. and Kwiatkowski, J., 1994. Developmental phonological disorders I: A clinical profile. https://doi.org/10.1044/jshr.3705.1100
6) Broomfield, J. and Dodd, B., 2004. Children with speech and language disability: caseload characteristics. https://doi.org/10.1080/13682820310001625589
7) Hayiou‐Thomas, M.E., Carroll, J.M., Leavett, R., Hulme, C. and Snowling, M.J., 2017. When does speech sound disorder matter for literacy? The role of disordered speech errors, co‐occurring language impairment and family risk of dyslexia. https://doi.org/10.1111/jcpp.12648
8) Lewis, B.A., Freebairn, L., Tag, J., Ciesla, A.A., Iyengar, S.K., Stein, C.M. and Taylor, H.G., 2015. Adolescent outcomes of children with early speech sound disorders with and without language impairment. https://doi.org/10.1044/2014_AJSLP-14-0075
9) Wren, Y., Miller, L.L., Peters, T.J., Emond, A. and Roulstone, S., 2016. Prevalence and predictors of persistent speech sound disorder at eight years old: Findings from a population cohort study. https://doi.org/10.1044/2015_JSLHR-S-14-0282
10) Johnson, C.J., Beitchman, J.H. and Brownlie, E.B., 2010. Twenty-year follow-up of children with and without speech-language impairments: Family, educational, occupational, and quality of life outcomes. https://doi.org/10.1044/1058-0360(2009/08-0083)
11) Le, H.N., Mensah, F., Eadie, P., McKean, C., Sciberras, E., Bavin, E.L., Reilly, S. and Gold, L., 2021. Health‐related quality of life of children with low language from early childhood to adolescence: Results from an Australian longitudinal population‐based study. https://doi.org/10.1111/jcpp.13277
12) St Clair, M.C., Forrest, C.L., Yew, S.G.K. and Gibson, J.L., 2019. Early risk factors and emotional difficulties in children at risk of developmental language disorder: A population cohort study. https://doi.org/10.1044/2018_JSLHR-L-18-0061
13) Burgoyne, K., Lervag, A., Malone, S. and Hulme, C., 2019. Speech difficulties at school entry are a significant risk factor for later reading difficulties. https://doi.org/10.1016/j.ecresq.2019.06.005

14) Pennington, B.F. and Bishop, D.V., 2009. Relations among speech, language, and reading disorders. https://doi.org/10.1146/annurev.psych.60.110707.163548
15) Royal College of Speech and Language Therapists (RCSLT), 2020. RCSLT briefing paper on Language Disorder with a specific focus on Developmental Language Disorder https://www.rcslt.org/wp-content/uploads/media/docs/Covid/language-disorder-briefing-paper-with-edit.pdf?la=en&hash=98B6A1E60824DEE9D52CCDFFACCE5EE6D67749D9
16) Dodd, B., 2014. Differential diagnosis of pediatric speech sound disorder. https://doi.org/10.1007/s40474-014-0017-3
17) Petinou, K., Taxitari, L., Phinikettos, I. and Theodorou, E., 2021. Dynamic linguistic interconnectedness and variability in toddlers. https://doi.org/10.1007/s10936-020-09747-y
18) Stoel-Gammon, C., 2011. Relationships between lexical and phonological development in young children. https://doi.org/10.1017/S0305000910000425
19) Ainsworth, S., Welbourne, S. and Hesketh, A., 2016. Lexical restructuring in preliterate children: Evidence from novel measures of phonological representation. https://doi.org/10.1017/S0142716415000338
20) Pascoe, M., Stackhouse, J., and Wells, B. Persisting Speech Difficulties in Children: Children's Speech and Literacy Difficulties. Chichester, UK: Whurr Publishers limited 2006: page 383
21) Tyler, A.A., 2002. Language-based intervention for phonological disorders. doi: 10.1055/s-2002-23511
22) Roulstone, S.E., Marshall, J.E., Powell, G.G., Goldbart, J., Wren, Y.E., Coad, J., Daykin, N., Powell, J.E., Lascelles, L., Hollingworth, W. and Emond, A., 2015. Evidence-based intervention for preschool children with primary speech and language impairments: Child Talk–an exploratory mixed-methods study. https://doi.org/10.3310/pgfar03050
23) Frizelle, P. and McKean, C., 2022. Using Theory to Drive Intervention Efficacy: The Role of Dose Form in Interventions for Children with DLD. https://doi.org/10.3390/children9060859
24) Rodgers, L., Harding, S., Rees, R. and Clarke, M.T., 2022. Interventions for pre-school children with co-occurring phonological speech sound disorder and expressive language difficulties: A scoping review. https://doi.org/10.1111/1460-6984.12719
25) Almost, D.A. and Peter Rosenbaum, M.D., 1998. Effectiveness of speech intervention for phonological disorders: A randomized controlled trial. https://doi.org/10.1111/j.1469-8749.1998.tb15383.x
26) Fong, N.W.Y., Ho, S.K.Y., So, B.J.W. and Lian, W.B., 2012. Evaluation of the Hanen it takes two to talk intervention programme. https://doi.org/10.1177/201010581202100406
27) McLeod, S., Baker, E., McCormack, J., Wren, Y., Roulstone, S., Crowe, K., Masso, S., White, P. and Howland, C., 2017. Cluster-randomized controlled trial evaluating the effectiveness of computer-assisted intervention delivered by educators for children with speech sound disorders. https://doi.org/10.1044/2017_JSLHR-S-16-0385
28) Sugden, E., Baker, E., Williams, A.L., Munro, N. and Trivette, C.M., 2020. Evaluation of parent-and speech-language pathologist–delivered multiple oppositions intervention for children with phonological impairment: A multiple-baseline design study. https://doi.org/10.1044/2019_AJSLP-18-0248

29) West, G., Snowling, M.J., Lervåg, A., Buchanan-Worster, E., Duta, M., Hall, A., McLachlan, H. and Hulme, C., 2021. Early language screening and intervention can be delivered successfully at scale: evidence from a cluster randomized controlled trial. https://doi.org/10.1111/jcpp.13415
30) Morgan, L., Marshall, J., Harding, S., Powell, G., Wren, Y., Coad, J. and Roulstone, S., 2019. ‘It depends’: Characterizing speech and language therapy for preschool children with developmental speech and language disorders. https://doi.org/10.1111/1460-6984.12498
31) Romeo, R.R., Leonard, J.A., Robinson, S.T., West, M.R., Mackey, A.P., Rowe, M.L. and Gabrieli, J.D., 2018. Beyond the 30-million-word gap: Children’s conversational exposure is associated with language-related brain function. https://doi.org/10.1177/0956797617742725
32) Morris C, Simkiss D, Busk M, *et al.*Setting research priorities to improve the health of children and young people with neurodisability: a British Academy of Childhood Disability-James Lind Alliance Research Priority Setting Partnership. doi: 10.1136/bmjopen-2014-006233
33) Dodd, B., 2014. Differential diagnosis of pediatric speech sound disorder. https://doi.org/10.1007/s40474-014-0017-3
34) Rodgers, L., and Harford., D. Collaborating with parents to identify outcomes for a preschool speech and language intervention. ESLA Congress 2022: New frontiers in speech and language therapy: advancing practice, research and education. DOI:10.13140/RG.2.2.34626.45763
https://www.researchgate.net/publication/362910019_Collaborating_with_parents_to_identify_outcomes_for_a_pre-school_speech_and_language_intervention
35) Rodgers, L., and Harford., D. 2022. Part of the family. RCSLT Bulletin. Issue 832, autumn 2022.
36) Zimmerman, I. L., Steiner, V. G., & Pond, R. E. (2002). *Preschool Language Scale, Fourth Edition (PLS-4)*.https://doi.org/10.1037/t15140-000
37) McLeod, S., Harrison, L.J. and McCormack, J., 2012. The intelligibility in context scale: Validity and reliability of a subjective rating measure. https://doi.org/10.1044/1092-4388(2011/10-0130)
38) Curran, M. & Owen Van Horne, A., 2019. Use of Recast Intervention to Teach Causal Adverbials to Young Children With Developmental Language Disorder Within a Science Curriculum: A Single Case Design Study. https://doi.org/10.1044/2018_AJSLP-17-0164
39) Miller, N., 2013. Measuring up to speech intelligibility. https://doi.org/10.1111/1460-6984.12061
40) Pommée, T., Balaguer, M., Mauclair, J., Pinquier, J. and Woisard, V., 2022. Intelligibility and comprehensibility: A Delphi consensus study. https://doi.org/10.1111/1460-6984.12672
41) Wren, Y., Harding, S., Goldbart, J. and Roulstone, S., 2018. A systematic review and classification of interventions for speech-sound disorder in preschool children. https://doi.org/10.1111/1460-6984.12371
42) Frizelle, P., Tolonen, A.K., Tulip, J., Murphy, C.A., Saldana, D. and McKean, C., 2021. The influence of quantitative intervention dosage on oral language outcomes for children with developmental language disorder: A systematic review and narrative synthesis. https://doi.org/10.1044/2020_LSHSS-20-00058

For peer review only

43) Carson, L., Baker, E. and Munro, N., 2022. A Systematic Review of Interventions for Late Talkers: Intervention Approaches, Elements, and Vocabulary Outcomes. https://doi.org/10.1044/2022_AJSLP-21-00168
44) Murray, E., Power, E., Togher, L., McCabe, P., Munro, N. and Smith, K., 2013. The reliability of methodological ratings for speechBITE using the PEDro-P scale. https://doi.org/10.1111/1460-6984.12007
45) Tate, R.L., Perdices, M., Rosenkoetter, U., Wakim, D., Godbee, K., Togher, L. and McDonald, S., 2013. Revision of a method quality rating scale for single-case experimental designs and n-of-1 trials: The 15-item Risk of Bias in N-of-1 Trials (RoBiNT) Scale. https://doi.org/10.1080/09602011.2013.824383
46) Law, J., Charlton, J., Dockrell, J., Gascoigne, M., McKean, C. and Theakston, A., 2017. Early Language Development: Needs, provision and intervention for pre-school children from socio-economically disadvantaged backgrounds. London: Education Endowment Foundation. https://educationendowmentfoundation.org.uk/public/files/Law_et_al_Early_Language_Development_final.pdf
47) Warren, S.F., Fey, M.E. & Yoder, P.J. 2007. Differential treatment intensity research: A missing link to creating optimally effective communication interventions. https://doi.org/10.1002/mrdd.20139
48) Hoffmann, T.C., Glasziou, P.P., Boutron, I., Milne, R., Perera, R., Moher, D., Altman, D.G., Barbour, V., Macdonald, H., Johnston, M. and Lamb, S.E., 2014. Better reporting of interventions: template for intervention description and replication (TIDieR) checklist and guide. https://doi.org/10.1136/bmj.g1687
49) Campbell, M., McKenzie, J.E., Sowden, A., Katikireddi, S.V., Brennan, S.E., Ellis, S., Hartmann-Boyce, J., Ryan, R., Shepperd, S., Thomas, J. and Welch, V., 2020. Synthesis without meta-analysis (SWiM) in systematic reviews: reporting guideline. https://doi.org/10.1136/bmj.l6890
50) Staniszewska, S., Brett, J., Simera, I., Seers, K., Mockford, C., Goodlad, S., Altman, D.G., Moher, D., Barber, R., Denegri, S. and Entwistle, A., 2017. GRIPP2 reporting checklists: tools to improve reporting of patient and public involvement in research. https://doi.org/10.1136/bmj.j3453

SUPPLEMENTARY MATERIALS 1:
DRAFT SEARCH STRATEGY FOR MEDLINE COMPLETE

Search date: 2/12/2022
Platform: EBSCOhost

Search ID#	Search Terms	Search Options	Actions
S1	AB (paediatric or pediatric or children or child or infant or infants or schoolchild or schoolchildren or preschool or "early years" or kindergarten) NOT MJ (teenage or teenagers or adolescent or adolescents or "young adults")	**Limiters** - Date of Publication: 20120101-; English Language<br>**Expanders** - Apply related words; Apply equivalent subjects<br>**Search modes** - Boolean/Phrase	View Results (719,273) View Details Edit
S2	AB ("speech delay" or "speech disorder" or "speech sound disorder" or phonological or phonology or "speech intelligibility" or "intelligible speech" or "speech comprehensibility" or "comprehensible speech" or articulation) NOT MJ ("sign language" or "mental retardation" or autism or "autistic spectrum disorder" or Asperger or "cleft lip" or "cleft palate" or deaf or "cerebral palsy" or aphonia or geriatrics)	**Limiters** - Date of Publication: 20120101-; English Language<br>**Expanders** - Apply related words; Apply equivalent subjects<br>**Search modes** - Boolean/Phrase	View Results (13,378) View Details Edit
S3	AB ("language delay" or "language disorder" or "specific language impairment" or "language impairment" or vocabulary or "word learning" or "language difficulties" or "developmental language disorder" or "late talker") NOT MJ ("sign language" or "mental retardation" or autism or "autistic spectrum disorder" or Asperger or "cleft palate" of "cleft lip" or deaf or "cerebral palsy" or aphonia or geriatrics)	**Limiters** - Date of Publication: 20120101-; English Language<br>**Expanders** - Apply related words; Apply equivalent subjects<br>**Search modes** - Boolean/Phrase	View Results (10,677) View Details Edit
S4	AB therapy or intervention or interventions or treatment or treatments or programme or programmes or program or programs or teaching or instruction or approach or approaches or technique or techniques or strategy or strategies or activity or activities or class or classes	**Limiters** - Date of Publication: 20120101-; English Language<br>**Expanders** - Apply related words; Apply equivalent subjects<br>**Search modes** - Boolean/Phrase	View Results (6,084,546) View Details Edit
S5	S2 OR S3	**Expanders** - Apply equivalent subjects<br>**Search modes** - Boolean/Phrase	View Results (23,007) View Details Edit
S6	S1 AND S5	**Expanders** - Apply equivalent subjects<br>**Search modes** - Boolean/Phrase	View Results (8,110) View Details Edit
S7	S4 AND S6	**Expanders** - Apply equivalent subjects<br>**Search modes** - Boolean/Phrase	View Results (3,264) View Details Edit

SUPPLEMENTARY MATERIALS 2: EXCLUSION SCREENING GUIDANCE

Population

Inclusion

80% children aged 2:0 and 5:11; idiopathic phonological speech production difficulties and/or difficulties relating to oral vocabulary; difficulties identified on standardised assessments, parental/and or professional observation reports, and/or pre-intervention baseline probes; idiopathic speech/language needs

Exclusion

Children with typically developing speech/language skills; speech/language difficulties not caused by or associated with a condition with a known impact on communication e.g. autism, deafness, cerebral palsy, cleft lip/palate, dysarthria

Title/abstract level specific: still include if any of the exclusion criteria remains unknown and the inclusion criteria is met or unknown.

Intervention

Inclusion: Any setting, deliverer, mode of delivery

Exclusion: N/A

Comparator

Inclusion: Empirical evaluation of intervention effectiveness from RCTs, experimental and quasi-experimental studies, case studies/within groups designs.

Exclusion: Assessment at a single timepoint pre and post intervention, with no comparator.

Title/abstract level specific: still include if any of the exclusion criteria remains unknown and the inclusion criteria is met or unknown.

Outcome

Inclusion: Measure of oral vocabulary and/or speech comprehensibility. Include intelligibility measures as a proxy for comprehensibility. Include proximal measures of vocabulary development that may arise from syntactic assessments, such as the number of different words (NDW). Outcomes must be evaluated via standardised assessment, probes, and/or observational ratings or scales. Post intervention assessment at any timepoint.

Exclusion: Composite measures where individual results for speech comprehensibility/intelligibility and/or oral vocabulary are not completed in a separate analysis. Purely syntactic measures of change, such as the mean length of utterance in morphemes (MLUm).

Title/abstract level specific: still include if any of the exclusion criteria remains unknown and the inclusion criteria is met or unknown. Still include if primary outcomes do not relate to

For peer review only

vocabulary/speech comprehensibility, but where the inclusion criteria are either met or unknown.

For peer review only

SUPPLEMENTARY MATERIALS 3:
DRAFT CONTENT FOR THE DATA EXTRACTION FORM

1. **General information/study details**
    - Date
    - Title
    - Location (country)
    - Language the intervention was in
    - Study design
    - Comparator
    - No. participants (incl. Participants in control/alternative experimental group if relevant)
    - Why- goals/aims of the overall intervention
    - Outcome/s measured of relevance to this review
    - Is this a primary outcome of the intervention?

2. **Population characteristics**
    - Age
    - Male/female
    - Languages spoken
    - Ethnicity
    - SES details as reported (e.g. parental education/employment)
    - Pre-intervention speech assessment levels
    - Pre-intervention language assessment levels (comprehension as well as expressive)

3. **Intervention characteristics**
    - Setting (e.g. home, nursery/school, clinic)
    - Was the intervention modified at any point? If so, how?
    - Was the intervention adapted/tailored to individual children at any point? If so, how?
    - Techniques within the intervention
    - Rationale for each technique/the technique within the wider approach
    - Mode of delivery of the technique (e.g. fully face to face in clinic; hybrid in clinic with some carryover over home and/or nursery; virtual delivery)
    - Was technique delivery implicit (e.g. listening to an adults' model) or explicit (e.g. being asked to repeat)?
    - The wider activity/game the technique is part of (e.g. shared book reading, child led play, everyday routines, a combination of these)
    - Dosage
        - Dose frequency of the individual technique (no. times delivered per session, day, across a week)
        - Dose frequency of the intervention as a whole
        - Total duration of the technique (the time period between which the technique is used)

For peer review only

- Total duration of the intervention (the time period/duration of the intervention as a whole)

  - Deliverer/s of the technique
  - If not SLT, how the person was trained to deliver the technique
  - Materials used to carry out the technique
  - Intervention fidelity measured? Report on this if so

4. Outcomes

- Measure/s used
- Timepoints
- Reported effect

For peer review only

PRISMA-P (Preferred Reporting Items for Systematic review and Meta-Analysis Protocols) 2015 checklist: recommended items to address in a systematic review protocol*

Section and topic	Item No	Checklist item	Location
**ADMINISTRATIVE INFORMATION**			
Title:			
Identification	1a	Identify the report as a protocol of a systematic review	Page 1 (title page)
Update	1b	If the protocol is for an update of a previous systematic review, identify as such	Page 2 (abstract) further details on page 4 (broader context: an intervention development study)
Registration	2	If registered, provide the name of the registry (such as PROSPERO) and registration number	Page 2 (abstract)
Authors:			
Contact	3a	Provide name, institutional affiliation, e-mail address of all protocol authors; provide physical mailing address of corresponding author	Page 1 (title page)
Contributions	3b	Describe contributions of protocol authors and identify the guarantor of the review	Page 16
Amendments	4	If the protocol represents an amendment of a previously completed or published protocol, identify as such and list changes; otherwise, state plan for documenting important protocol amendments	Page 2 (abstract); further details on page 8 (search strategy)
Support:			
Sources	5a	Indicate sources of financial or other support for the review	Page 16
Sponsor	5b	Provide name for the review funder and/or sponsor	Page 16
Role of sponsor or funder	5c	Describe roles of funder(s), sponsor(s), and/or institution(s), if any, in developing the protocol	Page 16
**INTRODUCTION**			
Rationale	6	Describe the rationale for the review in the context of what is already known	Pages 3-5 (introduction)
Objectives	7	Provide an explicit statement of the question(s) the review will address with reference to participants, interventions, comparators, and outcomes (PICO)	Page 5 (objectives and research questions); further details on pages 6/7 (eligibility criteria)
**METHODS**			
Eligibility criteria	8	Specify the study characteristics (such as PICO, study design, setting,	Pages 6/7 (eligibility criteria and information sources)

For peer review only

		time frame) and report characteristics (such as years considered, language, publication status) to be used as criteria for eligibility for the review	
Information sources	9	Describe all intended information sources (such as electronic databases, contact with study authors, trial registers or other grey literature sources) with planned dates of coverage	Page 7 (information sources)
Search strategy	10	Present draft of search strategy to be used for at least one electronic database, including planned limits, such that it could be repeated	Supplementary material document 1
Study records:			
Data management	11a	Describe the mechanism(s) that will be used to manage records and data throughout the review	Page 8
Selection process	11b	State the process that will be used for selecting studies (such as two independent reviewers) through each phase of the review (that is, screening, eligibility and inclusion in meta-analysis)	Pages 8/9
Data collection process	11c	Describe planned method of extracting data from reports (such as piloting forms, done independently, in duplicate), any processes for obtaining and confirming data from investigators	Pages 8/9, supplementary material document 3
Data items	12	List and define all variables for which data will be sought (such as PICO items, funding sources), any pre-planned data assumptions and simplifications	Pages 9/10, supplementary material document 2
Outcomes and prioritization	13	List and define all outcomes for which data will be sought, including prioritization of main and additional outcomes, with rationale	Page 10 (outcomes and prioritisation)
Risk of bias in individual studies	14	Describe anticipated methods for assessing risk of bias of individual studies, including whether this will be done at the outcome or study level, or both; state how this information will be used in data synthesis	Page 9 (risk of bias/internal validity); page 10 (risk of bias in individual studies); page 10 (quantitative data)
Data synthesis	15a	Describe criteria under which study data will be quantitatively synthesised	Page 10 (data synthesis)
	15b	If data are appropriate for quantitative synthesis, describe planned summary measures, methods of handling data and methods of combining data from studies, including any planned exploration of consistency (such as I^2, Kendall's τ)	Not applicable
	15c	Describe any proposed additional analyses (such as sensitivity or subgroup analyses, meta-regression)	Not applicable
	15d	If quantitative synthesis is not appropriate, describe the type of summary planned	Page 10 (data synthesis)
Meta-bias(es)	16	Specify any planned assessment of meta-bias(es) (such as publication	Page 11 (meta-bias)

For peer review only

		bias across studies, selective reporting within studies)	
Confidence in cumulative evidence	17	Describe how the strength of the body of evidence will be assessed (such as GRADE)	Page 11 (strength of body of evidence)

*** It is strongly recommended that this checklist be read in conjunction with the PRISMA-P Explanation and Elaboration (cite when available) for important clarification on the items. Amendments to a review protocol should be tracked and dated. The copyright for PRISMA-P (including checklist) is held by the PRISMA-P Group and is distributed under a Creative Commons Attribution Licence 4.0.**

From: Shamseer L, Moher D, Clarke M, Ghersi D, Liberati A, Petticrew M, Shekelle P, Stewart L, PRISMA-P Group. Preferred reporting items for systematic review and meta-analysis protocols (PRISMA-P) 2015: elaboration and explanation. BMJ. 2015 Jan 2;349(jan02 1):g7647.

For peer review only

The shared characteristics of intervention techniques for oral vocabulary and speech comprehensibility in pre-school children with co-occurring features of developmental language disorder and a phonological speech sound disorder: protocol for a systematic review with narrative synthesis

Journal:	*BMJ Open*
Manuscript ID	bmjopen-2022-071262.R1
Article Type:	Protocol
Date Submitted by the Author:	20-Mar-2023
Complete List of Authors:	Rodgers, Lucy; City University of London, Language and Communication Science; Sussex Community NHS Foundation Trust, Children's Speech and Language Therapy<br>Botting, Nicola ; City University of London, Department of Language and Communication Science<br>Cartwright, Martin; City University of London, Department of Health Services Research and Management<br>Harding, Sam; North Bristol NHS Trust, Bristol Speech and Language Therapy Research Unit<br>Herman, Rosalind; City University of London, Department of Language and Communication Science
**Primary Subject Heading**:	Paediatrics
Secondary Subject Heading:	Communication
Keywords:	Community child health < PAEDIATRICS, Speech pathology < OTOLARYNGOLOGY, Developmental neurology & neurodisability < PAEDIATRICS

TITLE PAGE

TITLE:
The shared characteristics of intervention techniques for oral vocabulary and speech comprehensibility in pre-school children with co-occurring features of developmental language disorder and a phonological speech sound disorder: protocol for a systematic review with narrative synthesis

AUTHORS:
Corresponding author: Lucy Rodgers. Department of Language and Communication Science, Myddelton Street Building, City University of London, EC1V 0HB. Lucy.rodgers@city.ac.uk https://orcid.org/0000-0001-6585-7206

Co-author: Prof. Nicola Botting. Department of Language and Communication Science, City University, London, UK.

Co-author: Dr Martin Cartwright. Department of Health Services Research and Management, City University, London, UK.

Co-author: Dr Sam Harding. Bristol Speech and Language Therapy Research Unit, North Bristol NHS Trust, Bristol, UK. https://orcid.org/0000-0002-5870-2094

Co-author: Prof. Rosalind Herman. Department of Language and Communication Science, City University, London, UK. http://orcid.org/0000-0001-5732-9999

Word count (excluding title page/abstract/references): 4,527

Key words: Language Development Disorders; Speech Sound Disorder; Child Language; Speech-Language Pathology

For peer review only

ABSTRACT

Introduction:
Evidence suggests that over a third of young children with Developmental Language Disorder (DLD) or Speech Sound Disorder (SSD) have co-occurring features of both. A co-occurring DLD and SSD profile is associated with negative long-term outcomes relating to communication, literacy and emotional wellbeing. However, the best treatment approach for young children with this profile is not understood. The aim of the proposed review is to identify intervention techniques for both DLD and SSD, along with their shared characteristics. The findings will then be analysed in the context of relevant theory. This will inform the content for a new or adapted intervention for these children.

Methods and analysis:
This search will build on a previous systematic review by Roulstone et al. (2015) but with a specific focus on oral vocabulary (DLD outcome) and speech comprehensibility (SSD outcome). These outcomes were identified by parents and Speech and Language Therapists within the pre-study stakeholder engagement work. The following databases will be searched for articles from January 2012 onwards: Ovid EMCare, Medline Complete, CINAHL, APA PsychINFO, Communication Source and ERIC. Two reviewers will independently perform the title/abstract screening and the full text screening with the exclusion criteria document being revised in an iterative process. Articles written in languages other than English will be excluded. Data will be extracted regarding key participant and intervention criteria, including technique dosage and delivery details. This information will then be pooled into a structured narrative synthesis.

Ethics and dissemination: Ethical approval is not needed for a systematic review protocol. Dissemination of findings will be through peer-reviewed publications, social media, and project steering group networks.

Study registration number: PROSPERO, CRD4202237393

Strengths and limitations of this study

- This protocol follows the referred Reporting Items for Systematic Review and Meta-Analysis Protocols (PRISMA-P) guidelines.
- Electronic databases spanning medicine, education, and psychology will be searched.
- Electronic databases in languages other than English will not be searched.
- Meta-bias(es) within the literature cannot be fully controlled.
- The level of detail within intervention reporting, as per the TIDieR guidelines, has the potential to vary amongst studies.

For peer review only

INTRODUCTION

Within the field of child language disorders, there are often overlapping or co-occurring difficulties which create unique patient experiences. Yet, while there is ample literature on treatment for singly occurring difficulties, there is a notable gap in evidence for treating children with co-occurring disorders. This review focusses on intervention for children who have co-occurring features of both Developmental Language Disorder (DLD) and Speech Sound disorder (SSD).

Co-occurring Developmental Language Disorder (DLD) and Speech Sound Disorder (SSD)

An estimated 7.58% of 4 year olds present with features of a Developmental Language Disorder (DLD)(1). DLD is characterised by idiopathic difficulties in using and understanding spoken language (2). One feature is limited vocabulary development (2), which has a known association with childhood temper tantrums/mental health, and later language and literacy skills (3,4). Such features of DLD may co-occur with a Speech Sound Disorder (SSD); that is, difficulties in producing speech sounds (5). An estimated 3.4% of 4- year olds have SSD (6). One of the most devastating impacts SSD is the impact on a child's ability to make themselves understood to others in everyday life (7). The term for this is speech comprehensibility (8). A related term, speech intelligibility, refers to the acoustic-phonetic decoding of utterances, and is very closely related to speech comprehensibility as both are linked to the functional use of speech. As with limited vocabulary, poor speech comprehensibility/intelligibility within the early years have also been associated with negative longer-term outcomes, including persisting speech difficulties (9) and poor literacy skills (10,11). Although it is typical for very young children not to be fully understood to those around them as their speech develops, by 4 years of age a child would typically be at least 50% intelligible (12).

Thirty six percent of 4-year olds with idiopathic SSD also have oral (i.e. expressive-spoken) language features of DLD (6). This high rate of co-occurrence is in keeping with historical research in the area (13), as well as study data from clinical caseloads (14). The combined impact of co-occurring features of DLD/SSD is twofold; for example, for a child with limited oral vocabulary and speech comprehensibility, not only are they unable to use many words, but the limited words they do have will not be understood to others within their daily lives. It therefore may be unsurprising that co-occurring phonological DLD/SSD features in early childhood are associated with negative long-term outcomes relating to literacy (15,16) and communication (17,18), with downstream consequences for quality of life (18,19) and emotional wellbeing (20). Consequently, access to effective and appropriately targeted intervention for children with this profile is crucial.

Phonological SSDs are the most frequently presenting SSD subtype (5), and occur when a child has difficulties with manipulating the different sound contrasts (phonemes) which are needed to form words (21). There are different types of phonological SSDs, including consistent phonological disorder (where the child makes consistent sound omissions or substitutions) and inconsistent phonological disorder (where these errors have no consistent pattern) (21). Research highlights a known link between DLD and phonological SSDs, as both disorders are underpinned by shared linguistic deficits (2). This overlap is

For peer review only - http://bmjopen.bmj.com/site/about/guidelines.xhtml

represented in the seminal CATALISE DLD consensus paper (2). In contrast to phonological SSDs, the CATALISE authors' speech, language and communication needs diagram highlights how other SSD subtypes, such as motor based SSDs like dysarthria, have a less marked overlap with DLD. They also often have a known cause (i.e. are non-idiopathic). Due to their significant overlap with DLD which has no known causation, this review will focus on phonological SSDs which are also idiopathic in nature.

Figure 1: DLD within the broader category of Speech, Language and Communication Needs (SLCN) (2)

The overlap between language and phonological SSDs is further supported by studies on the speech and language development of young children, where complex and bi-directional relationships between the development of individual sounds (phonology) and words (the lexicon) have been identified (22,23). For example, the first words of young children primarily consist of the speech sounds already established within their emerging phonological inventory (23). This relationship between phonology and the lexicon may have implications for intervention with children with co-occurring features of DLD and a phonological SSD. For example, growth in vocabulary and/or the strengthening of phonological representations has the potential to impact speech and vocabulary development concurrently through a process known as 'lexical restructuring' (24). A further psycholinguistic theory of potential relevance is the speech processing model (25), which suggests that individual children with co-occurring features of DLD/a phonological SSD may have difficulties at one or more levels of speech processing, rather than just with phonological representations alone. Such theories are important within interventions for co-occurring DLD/phonological SSD as they can be used to inform intervention content and delivery.

Current interventions for pre-school co-occurring DLD/SSD

Although this overlap exists between DLD and phonological SSDs, there is currently a paucity of theoretically informed interventions which have been specifically developed for this group (26). Additionally, intervention studies within existence primarily target morphosyntactic aspects of expressive language, alongside accuracy of speech sound production (26,27). However, for younger children with this profile, and children whose features of DLD are more severe, building vocabulary is typically targeted in speech and language therapy prior to morphosyntax (28).

'Child Talk' (29) was a large National Institute of Health Research (NIHR) funded mixed methods programme of work, including a systematic review. This involved investigating the use of early years' speech and language therapy interventions. The findings led to the specification of a) a typology of early years' speech and language therapy (SLT) intervention, b) key intervention ingredients for each typology theme. A technique can be described as "the specific teaching behaviours/actions thought to effect change" (30). The findings highlighted that for children with co-occurring features of DLD/SSD, clinicians often adapt existing interventions by selecting and combining different techniques. This enables them to use their knowledge and experience to provide the best treatment that they can (29,31).

For peer review only - http://bmjopen.bmj.com/site/about/guidelines.xhtml

Although our knowledge of what techniques work best for children with this profile is limited, techniques identified might be related to underlying theories of potential relevance. For example, language modelling is typically linked to growth in expressive language (32). However, based on the lexical restructuring hypothesis, it is hypothesised that the subsequent impact of this language growth on the accuracy and segmentation of the child's phonological representations could also influence their phonological speech sound production (24). 'Building things into play' was also a key technique highlighted in Child Talk. Theoretically, this is supported by the latest research on the brain basis of speech and language learning, which indicates that learning best takes place within interactions which are meaningful for the child (33,34). Considering this, it is hypothesised that this technique supports speech and language learning through capitalising on the child's heightened attention and motivation during the play activity.

These considerations highlight a valuable opportunity for an intervention specific to this clinical group to be developed, utilising techniques which can be supported by relevant theory. Due to the current paucity of evidence, the associated negative impact of this co-occurring profile on long term outcomes, and the high level of presentation on clinical caseloads, there is an urgent need for such intervention development to take place. The first stage in this development would be to conduct a systematic review to identify potential techniques of relevance.

Broader context: an intervention development study

The proposed review updates the systematic review findings from 'Child Talk' (29) whilst refining the focus to techniques within interventions for children with features of DLD or a phonological SSD. Techniques will be extracted from included studies and then analysed in relation to shared characteristics and underpinning theory. The synthesis will then be used to inform the content of a new intervention which is being developed for young children with co-occurring features of DLD/phonological SSD.

Both DLD and SSD are heterogenous disorders (2,21), and therefore have a range of associated outcomes. This review, and body of intervention development work it is a part of, will focus exclusively on the outcomes of oral vocabulary (DLD outcome) and speech comprehensibility (SSD outcome). This is due to the afore mentioned impact of such difficulties on the everyday lives of young children; this decision is also elaborated on in the 'patient and public involvement' section of this paper.

Based on the dose form framework (30), shared characteristics for DLD/phonological SSD intervention techniques may include similarities in:

1) *who* delivers the technique; for example, is it the parent, clinician, or both?
2) *where* the technique is delivered; for example, at home, nursery, clinic or a combination of these?
3) the nature of *technique delivery*; for example, is it during an adult led structured game, child led play, everyday routines, or a combination of these?

Underpinning theory may relate to:

For peer review only

1)The lexical restructuring hypothesis (24)
2) Psycholinguistic models of speech and language development; such as the speech processing model (25)
3)The neural basis for speech and language development; for example, the role of meaningful interactions within language learning (33)

Objectives

The overarching aim of the review is to bring together intervention techniques from DLD and phonological SSD interventions. The objectives within this are to:

1. Identify the shared core characteristics of the techniques; this includes the deliverer, place of delivery, format of delivery and nature of delivery *(e.g. child or adult led)*

2. Compare and synthesise the shared core characteristics of the techniques in relation to underlying theory

3. Establish the best available evidence for interventions that incorporate these core characteristics of the intervention techniques

Research questions:

1)What are the shared core characteristics of intervention techniques in preschool interventions targeting speech comprehensibility and/or oral vocabulary?

2)How do these shared core characteristics relate to underlying theory?

3)What evidence is there for the effect of interventions that incorporate these core characteristics of intervention techniques?

METHODS AND ANALYSIS

In accordance with the guidelines, our systematic review protocol has been registered with the International Register of Systematic Reviews (PROSPERO) on 16/12/2022 (registration number CRD42022373931). In the event of any amendments to methodology set out below, the date of each amendment will be accompanied by a description of the change and the rationale in either the Prospero register and/or the final results paper.

Eligibility criteria

The eligibility criteria stated below are in line with the criteria from the original 'Child Talk' systematic review (29) with amendments according to the objectives of the current review. Most importantly, this review will focus specifically on the 'expressive language' and 'speech' themes generated from their initial typology of early years' Speech and Language

Therapy (SLT) interventions, as these themes encompass the two outcomes for which we are seeking to identify techniques.

Study designs

Included studies must report on an empirical evaluation of the effectiveness of an intervention. To ensure we identify all relevant literature, a range of study designs will be included. These include randomised control trials (RCTs), experimental and quasi-experimental studies, within subjects designs (e.g. pre-post studies) and case studies (which may include multiple baseline or other systematic manipulation of the intervention). Studies which report on single timepoint (e.g. cross-sectional studies) will be excluded. Studies focusing on efficacy, including lab-based training, will not be excluded if all other inclusion criteria are met. This is because information on the efficacy of speech/language learning techniques can be gleaned from these studies, although careful consideration will be given to how these results are integrated into the narrative analysis (further information on this is provided under 'data synthesis').

Population

To capture the age group most typically seen within clinical services, 80% of children within included studies must have been aged between 2:0 and 5:11 years. Additionally, although this review is part of a wider intervention development study for children aged 3 and 4 years, an expanded age range within this review will help to ensure all that techniques of potential relevance will be captured. The children within included studies must have presented with phonological speech production difficulties and/or difficulties relating to oral vocabulary, with all subtypes of phonological SSD included (e.g. consistent and inconsistent phonological disorder, phonological delay).These difficulties may be identified by standardised assessments such as the Preschool Language Scale (35), parental and/or professional observation reports such as the intelligibility in context scale (36) and/or probes. Probes may also be used to assess progress through the repeated measurement of the dependent variable before, during and after the intervention. As already observed in the literature, common probes within speech and language therapy interventions may include a selection of words containing the child's targeted speech sound/s or vocabulary (37,38). In keeping with the afore mentioned diagnostic description within CATALISE (2), included papers must state that the participants' needs had no obvious cause, i.e., excluding children with neurodevelopmental differences that have a known association with speech and/or language development, such as Autism or Cerebral Palsy. Due to the challenges in diagnosing DLD in very young children (2), and in order to maximise the identification of potentially relevant intervention techniques, studies will be included where the child does not have a formal diagnosis of DLD but is a late talker.

Intervention

We will include studies reporting on interventions delivered in any setting (e.g., home based, clinic) or format (e.g., face to face, online). The deliverer may be a speech and language therapist, speech and language therapy assistant, or equivalent professional (including education staff), and the intervention may involve professionals training up others (e.g., parents) to deliver some or all of the intervention.

For peer review only

Comparator

Comparators for included studies may be a control without an intervention (including multiple baseline and within subjects designs) or an alternative experimental group (i.e., intervention comparison).

Outcomes

Included papers must measure the effectiveness of the intervention on a) oral vocabulary, and/or b) speech comprehensibility. These outcomes must be evaluated via standardised assessment, probes, and/or observational ratings or scales.

If composite speech and language assessments are used, studies must report on the separate sub test results for oral vocabulary and/or speech comprehensibility to be included.

Studies with only syntactic measures of language change will be excluded; this includes mean length of utterance in morphemes (MLUm). However, they will be included if a proximal measure of vocabulary change is used alongside syntactic measures, such as the number of different words (NDW).

Speech comprehensibility is the SSD outcome in focus. As previously mentioned, comprehensibility and intelligibility are overlapping but differing constructs, with a shared focus on functional human communication (8). Therefore, we will also include studies with an outcome of improved speech intelligibility as a proxy for comprehensibility. This was deemed more suitable than using measures such as PCC as a proxy for comprehensibility, with their focus being on speech accuracy. Due to the very recent consensus in terminology, measures for comprehensibility might include measures with 'intelligibility' within their title, such as the 'intelligibility in context' scale, which is becoming increasingly utilised in SSD intervention research (36).

Information sources

The search will be conducted in Ovid EMCare, Medline Complete, CINAHL, APA PsychINFO, ERIC and Communication Source. These sources have been selected as they encompass the fields of health (medical, nursing and allied health professions), speech and language therapy, education and psychology and have been successfully utilised in previous reviews in the field (26,39). To support with literature saturation, supplementary search methods will be employed; this includes screening the reference lists from prominent reviews in the field post 2012 (40,41). We have selected reviews from 2012 onwards due to the original search going up to this date (29). Reference lists from included papers within the current search will also be screened for potential study eligibility. Forward citation searches in Web of Science (using the core collection) will also be carried out, with additional searches in Scopus if the titles are not available in Web of Science.

Due to resource constraints, articles written in languages other than English will be excluded. However, articles written in English where the participants speak languages other than English will be included. Additionally, grey literature searching will be confined to the inclusion of theses/dissertations, via the databases stated above. Thesis/dissertations have

For peer review only

been selected as although the original review (29) included a range of grey literature, thesis/dissertations were the only grey literature sources which contributed studies within the final included papers. In keeping with the original review, thesis/dissertations will only be included when a corresponding journal article cannot be found for the study.

Search strategy

Together with support from a specialist librarian, we will conduct an update of the original 'Child Talk' systematic review (29), searching articles from January 2012 to the present day. One of the researchers (SH) undertaking the current search also led on the original review. Relevant studies from the original 'speech' and 'expressive language' typology themes within the original 'Child Talk' review have already been located by reviewing the recorded outcomes for each study as stated on the original data extraction spreadsheet. Out of 41 papers from the 'speech' theme, two were found to address the outcome of comprehensibility/intelligibility. From the 30 papers within the 'expressive language' theme, 12 were found to include oral vocabulary as an outcome. These 14 papers will be further screened at stage 2 of the screening process (full text stage, outlined below).

The original review search strategy (29) has been updated for the current review, accounting for advances in terminology, e.g., consensus on the term 'Developmental Language Disorder' (2). The original 'Child Talk' search encompassed a broader range of speech and language outcomes, therefore the search terms for the current review have been adjusted to focus on our two specific outcomes of interest; oral vocabulary and speech comprehensibility. The updated search strategy was initially reviewed by two independent post-doctoral researchers in the field and adjusted as needed, for example, adding in the term 'specific language impairment', which may be relevant to older papers in the search. For the revised search strategy draft for each database, please see supplementary material 1.

Study records, Selection and data collection process

Search results will initially be imported into RefWorks, where duplicates will be removed by the first author (LR). The remaining articles will then be uploaded to the Covidence systematic review management database.

Initially, the first author (LR) will trial the exclusion guidance criteria document on 30 papers. For the initial draft of this exclusion criteria guidance document, please see supplementary material 2. The 30 papers will be randomly selected using a random number generator. These 30 papers will then be reviewed by a second reviewer. The reviewers will then meet to discuss discrepancies and make amendments to the exclusion guidance document if needed.

The screening and data extraction will be carried out as follows:

1) *Title/abstract screening*
   The full set of titles/abstracts will be screened by the first author (LR). If uncertainty arises about how to apply to eligibility criteria to a specific paper, these articles will

For peer review only

be discussed with a member of the review team (who is not involved in the formal screening process). This may then lead to further revisions to the exclusion criteria document. Following this, a second independent reviewer (SH) will independently screen the titles/abstracts. Any disagreements, and how these may relate to the exclusion guidance document, will be discussed in consensus meetings. Any disputed articles will then be re-screened should alterations have been made to the exclusion criteria document. If disagreement is not caused by confusion over the exclusion criteria document, and consensus cannot be reached through discussion, a third reviewer will be consulted.

2) *Full paper screening*
At the full text screening stage, two reviewers (LR, SH) will independently appraise all of the remaining articles for inclusion, following the iterative process as outlined for stage 1.

To enable transparency of the reliability of screening at stages 1 and 2, Cohen's K for these stages will be reported in the final paper.

3) *Risk of bias/internal validity*
Retained studies will then undergo assessment of internal validity by two independent reviewers (LR, SH). The reviewers will have regular consensus meetings, after independently assessing up to 4 papers at a time, to resolve potential conflicts. If disagreements persist, a third reviewer will be involved. Disagreements that arise (including those that have been resolved) will be recorded and reported in the final paper.

For the PEDro-P (42), papers with a rating of six and over will be retained for data extraction. This aligns with the original review (29). On the Risk of Bias in N-of-1 Trials (RoBiNT) scale, included studies will be rated as *fair* or above (43).

4) *Data extraction*
The first author (LR) will extract data from the first 25% of studies. These will be randomly selected using random number generation. A second extractor (SH) will then independently extract data from the same studies. The two extractors will then meet to discuss potential discrepancies, and to update the data extraction form if needed. Following this, the first author (LR) will extract the remainder of the data.

Data items

Data will be sought regarding general study information (e.g., date; study type; location; participant numbers), population characteristics (e.g., male/female; age; speech/language profile-including phonological SSD subtype), intervention techniques (e.g., dosage; underpinning theory and justification given by the authors) and reported impact on the outcome of interest. Data on reported participant SES background will also be obtained, due to this being a known risk factor within developmental speech and language disorders (44). We will also collate information on the number of languages spoken by the participants, as

For peer review only

well as reported ethnicities, with this being a potential factor for the external validity of findings (i.e., relevance to 'everyday' clinical practice).

The Template for Intervention Description and Replication (TIDieR) will be used as a framework to guide the extraction process (45), combined with the speech and language therapy specific 'Dose form Framework and Definitions', based on the work of Warren and colleagues (46); this has been applied in other reviews specific to paediatric speech and language therapy intervention (40). Details of techniques will be extracted regarding intervention contexts (e.g. the overarching activity the technique is presented in), method of instruction (e.g. who delivers the technique, where and when), and technique dosage (dose frequency and dose duration). All reported dosage information will be extracted in order to allow for variation in study design; most notably, studies which target both oral vocabulary and speech comprehensibility concurrently.

Outcomes and prioritisation

The two outcomes (oral vocabulary, speech comprehensibility) are of equal interest within this review, regardless of whether they are primary or secondary outcomes within the included studies.

Risk of bias in individual studies

Individual studies will be assessed for internal validity. To encompass the range of study designs included within this review, we will use the PEDro-P (42). Specifically, for single case experimental designs, the RoBiNT scale will be used (43).

Data synthesis

Quantitative data
Overarching details for each included study, including the individual internal validity ratings, will be given in the first table. Two summary graphs will also be presented to convey the percentage of overall ratings from the PEDro-P and RoBiNT Scales. The frequency of techniques within the included papers will be presented either numerically within a table, or within a graph or chart if this deemed more suited to the data collected. We will be guided by the synthesis without meta-analysis (SWiM) in systematic review guidelines (47) and will report on the direction of effect of the interventions, using vote counting with a sign test if appropriate.

Qualitative data
A description of the identified techniques will be presented in a table, including details regarding how they were operationalised, based on TIDieR (45) and the dose form framework (23).

The narrative synthesis will include sections on:
1)Similarities and differences (including shared core characteristics) between techniques used for the different outcomes
2)Patterns of technique dosage and delivery across the interventions

For peer review

3)How the similarities and differences (including shared core characteristics) in techniques relate to underlying theory. Depending on findings, this section will be broken down into sub sections focusing on each theory of interest, potentially including (but not necessarily limited to):
- the lexical restructuring hypothesis
- the speech processing model
- the neural basis for speech and language development
4)The effectiveness of interventions which contain these techniques/shared core characteristics of techniques

If relevant, any observed differences between interventions for different phonological SSD sub types will be incorporated into the narrative synthesis, or given in an additional section if deemed to be more appropriate to the data found.

In the event of lab-based training studies meeting the final inclusion criteria, this data will be presented on a separate table. Additionally, within the narrative synthesis itself they will not be directly compared to the effectiveness studies. Instead, they will be used to support any potential theory building arising from the synthesis.

If challenges are identified regarding gaps and quality in the knowledge base, this will also be explored within the results and discussion section of the corresponding results paper.

Patient and Public Involvement

According to the James Lind Alliance, knowing how to best select communication strategies according to a child's individual profile is the 2nd most important recommendation for research (48). This is strongly in keeping with the aims of this review, and highlights the broader relevance of this work.

For our wider intervention development study, outcomes were prioritised by clinicians and parents of pre-school children with DLD/SSD within pre-study PPIE work (49,50). They identified the outcomes of increasing 1) oral vocabulary *(DLD outcome)*, and 2) speech comprehensibility *(SSD outcome)*. This provides further support focusing on techniques that directly target oral vocabulary and speech comprehensibility.

In keeping with the integral role of PPIE throughout, a newly formed project PPIE steering group will provide input at key points in the review process. This is a diverse group consisting of parents, speech and language therapists, a person with DLD, a specialist early years educator, a bi/multi-lingual educational family support worker and a clinical equality, diversity and inclusion (EDI) expert. During the review, they will be involved with:

1) Reviewing the content for the data extraction form (supplementary document 3), prior to the data extraction phase

2) Identifying what data has the most relevance in the 'real world', with these potentially informing recommendations within the final paper

For peer review only - http://bmjopen.bmj.com/site/about/guidelines.xhtml

3) Defining and agreeing key messages to take from the review, and dissemination through the steering group networks.

Steering group input will be recorded and reported in the final article, in accordance with the GRIPP 2 reporting criteria short form (51).

Meta-bias (es)

It is important to acknowledge that meta-bias, including reporting and publication bias, is present within all aspects of health research. Although it is not possible to completely control for such bias, we will:

1) Establish if the protocol for each study was published before recruitment for participants commenced (where possible)

2) Compare the outcomes and results sections of the published report when a protocol is available (for when considering selective reporting bias)

3) Assess potential publication bias through the inclusion of prioritised grey literature (thesis/dissertations)

Confidence in Cumulative evidence

Confidence within the evidence as a whole will be based on the summary of the internal validity, as presented in the two summary tables (see data synthesis section). We will also acknowledge and discuss key factors relating to meta bias, and how the review findings should be interpreted based on this.

Figure 1: DLD within the broader category of Speech, Language and Communication Needs (SLCN) (2)

Ethics and dissemination:
As a systematic review this study does not warrant ethics board approval. Findings will be disseminated though peer reviewed publications, social media, and project steering group networks.

Registration details:
Study registration number: PROSPERO, CRD42022373931

Authors' contributions
LR led on this work and independently developed an initial draft of the manuscript (and appended documents). RH, NB, MC and SH suggested amendments after reviewing this and 3 subsequent re-drafts by LR. LR completed the fourth and final version of the manuscript, which was reviewed and agreed by all of the authors. LR led on the manuscript revisions following peer review, in collaboration with NB and SH. All authors agreed to the final submitted version.

For peer review only

The authors would also like to acknowledge and thank Dr Pauline Frizelle (University College Cork) and Dr Helen Stringer (Newcastle University) for providing expert independent reviews of the initial terms for the search strategy.

Competing interests' statement

There are no competing interests to declare.

Funding

This research was funded in whole, or in part, by the Wellcome Trust (Grant number 223500/Z/21/Z). For the purpose of open access, the author has applied a CC BY public copyright licence to any Author Accepted manuscript version arising from this submission.

For peer review only

FULL REFERENCES

1)Norbury, C.F., Gooch, D., Wray, C., Baird, G., Charman, T., Simonoff, E., Vamvakas, G. and Pickles, A., 2016. The impact of nonverbal ability on prevalence and clinical presentation of language disorder: Evidence from a population study. https://doi.org/10.1111/jcpp.12573

2)Bishop, D.V., Snowling, M.J., Thompson, P.A., Greenhalgh, T., Catalise-2 Consortium, Adams, C., Archibald, L., Baird, G., Bauer, A., Bellair, J. and Boyle, C., 2017. Phase 2 of CATALISE: A multinational and multidisciplinary Delphi consensus study of problems with language development: Terminology. https://doi.org/10.1111/jcpp.12721

3)Duff, F.J., Reen, G., Plunkett, K. and Nation, K., 2015. Do infant vocabulary skills predict school-age language and literacy outcomes?. Journal of Child Psychology and Psychiatry, 56(8), pp.848-856. https://doi.org/10.1111/jcpp.12378

4)Manning BL, Roberts MY, Estabrook R, Petitclerc A, Burns JL, Briggs-Gowan M, Wakschlag LS, Norton ES., 2019. Relations between toddler expressive language and temper tantrums in a community sample. Journal of Applied Developmental Psychology, volume 65, article 101070. https://doi.org/10.1016/j.appdev.2019.101070

5)Dodd, B., Reilly, S., Ttofari Eecen, K. and Morgan, A.T., 2018. Articulation or phonology? Evidence from longitudinal error data. https://doi.org/10.1080/02699206.2018.1488994

6)Eadie, P., Morgan, A., Ukoumunne, O.C., Ttofari Eecen, K., Wake, M. and Reilly, S., 2015. Speech sound disorder at 4 years: Prevalence, comorbidities, and predictors in a community cohort of children. https://doi.org/10.1111/dmcn.12635

7)McCormack J, McLeod S, McAllister L, Harrison LJ. My speech problem, your listening problem, and my frustration: The experience of living with childhood speech impairment. Speech, Language and hearing Services in Schools, *41*(4), pp.379-392. https://doi.org/10.1044/0161-1461(2009/08-0129)

8)Pommée, T., Balaguer, M., Mauclair, J., Pinquier, J. and Woisard, V., 2022. Intelligibility and comprehensibility: A Delphi consensus study. https://doi.org/10.1111/1460-6984.12672

9)To, C.K.S., McLeod, S., Sam, K.L. and Law, T., 2022. Predicting Which Children Will Normalize Without Intervention for Speech Sound Disorders. *Journal of Speech, Language, and Hearing Research*, *65*(5), pp.1724-1741. https://doi.org/10.1044/2022_JSLHR-21-00444

10) Burgoyne K, Lervag A, Malone S, Hulme C. 2019. Speech difficulties at school entry are a significant risk factor for later reading difficulties. Early Childhood Research Quarterly. *1* (49), pp. 40-8. https://doi.org/10.1016/j.ecresq.2019.06.005

11)Jin F, Schjølberg S, Wang MV, Eadie P, Nes RB, Røysamb E, Tambs K. 2020. Predicting literacy skills at 8 years from preschool language trajectories: A population-based cohort study. Journal of Speech, Language, and Hearing Research,*63*(8), pp. 2752-62. https://doi.org/10.1044/2020_JSLHR-19-00286

For peer review only - http://bmjopen.bmj.com/site/about/guidelines.xhtml

12)Hustad KC, Mahr TJ, Natzke P, Rathouz PJ. 2021. Speech development between 30 and 119 months in typical children I: Intelligibility growth curves for single-word and multiword productions. Journal of Speech, Language, and Hearing Research, *64*(10), pp.3707-19. https://doi.org/10.1044/2021_JSLHR-21-00142

13)Shriberg, L.D. and Kwiatkowski, J., 1994. Developmental phonological disorders I: A clinical profile. https://doi.org/10.1044/jshr.3705.1100

14)Broomfield, J. and Dodd, B., 2004. Children with speech and language disability: caseload characteristics. https://doi.org/10.1080/13682820310001625589

15)Hayiou-Thomas, M.E., Carroll, J.M., Leavett, R., Hulme, C. and Snowling, M.J., 2017. When does speech sound disorder matter for literacy? The role of disordered speech errors, co-occurring language impairment and family risk of dyslexia. https://doi.org/10.1111/jcpp.12648

16)Lewis, B.A., Freebairn, L., Tag, J., Ciesla, A.A., Iyengar, S.K., Stein, C.M. and Taylor, H.G., 2015. Adolescent outcomes of children with early speech sound disorders with and without language impairment. https://doi.org/10.1044/2014_AJSLP-14-0075

17)Wren, Y., Miller, L.L., Peters, T.J., Emond, A. and Roulstone, S., 2016. Prevalence and predictors of persistent speech sound disorder at eight years old: Findings from a population cohort study. https://doi.org/10.1044/2015_JSLHR-S-14-0282

18)Johnson, C.J., Beitchman, J.H. and Brownlie, E.B., 2010. Twenty-year follow-up of children with and without speech-language impairments: Family, educational, occupational, and quality of life outcomes. https://doi.org/10.1044/1058-0360(2009/08-0083)

19)Le, H.N., Mensah, F., Eadie, P., McKean, C., Sciberras, E., Bavin, E.L., Reilly, S. and Gold, L., 2021. Health-related quality of life of children with low language from early childhood to adolescence: Results from an Australian longitudinal population-based study. https://doi.org/10.1111/jcpp.13277

20)St Clair, M.C., Forrest, C.L., Yew, S.G.K. and Gibson, J.L., 2019. Early risk factors and emotional difficulties in children at risk of developmental language disorder: A population cohort study. https://doi.org/10.1044/2018_JSLHR-L-18-0061

21)Dodd, B., 2014. Differential diagnosis of pediatric speech sound disorder. https://doi.org/10.1007/s40474-014-0017-3

22)Petinou, K., Taxitari, L., Phinikettos, I. and Theodorou, E., 2021. Dynamic linguistic interconnectedness and variability in toddlers. https://doi.org/10.1007/s10936-020-09747-y

23)Stoel-Gammon, C., 2011. Relationships between lexical and phonological development in young children. https://doi.org/10.1017/S0305000910000425

For peer review only - http://bmjopen.bmj.com/site/about/guidelines.xhtml

24)Ainsworth, S., Welbourne, S. and Hesketh, A., 2016. Lexical restructuring in preliterate children: Evidence from novel measures of phonological representation. https://doi.org/10.1017/S0142716415000338

25)Pascoe, M., Stackhouse, J., and Wells, B. Persisting Speech Difficulties in Children: Children's Speech and Literacy Difficulties. Chichester, UK: Whurr Publishers limited 2006: page 383

26)Rodgers, L., Harding, S., Rees, R. and Clarke, M.T., 2022. Interventions for pre-school children with co-occurring phonological speech sound disorder and expressive language difficulties: A scoping review. https://doi.org/10.1111/1460-6984.12719

27)Tyler AA, Lewis KE, Haskill A, Tolbert LC. Efficacy and cross-domain effects of a morphosyntax and a phonology intervention. 2002. Language, Speech, and Hearing Services in Schools. https://doi.org/10.1044/0161-1461(2002/005)

28)Department for Education. 2012. Exploring interventions for children and young people with speech, language and communication needs: A study of practice. https://assets.publishing.service.gov.uk/government/uploads/system/uploads/attachment_data/file/219627/DFE-RR247-BCRP13.pdf

29)Roulstone, S.E., Marshall, J.E., Powell, G.G., Goldbart, J., Wren, Y.E., Coad, J., Daykin, N., Powell, J.E., Lascelles, L., Hollingworth, W. and Emond, A., 2015. Evidence-based intervention for preschool children with primary speech and language impairments: Child Talk–an exploratory mixed-methods study. https://doi.org/10.3310/pgfar03050

30)Frizelle, P. and McKean, C., 2022. Using Theory to Drive Intervention Efficacy: The Role of Dose Form in Interventions for Children with DLD. https://doi.org/10.3390/children9060859

31)Morgan, L., Marshall, J., Harding, S., Powell, G., Wren, Y., Coad, J. and Roulstone, S., 2019. 'It depends': Characterizing speech and language therapy for preschool children with developmental speech and language disorders. https://doi.org/10.1111/1460-6984.12498

32)Girolametto L, Pearce PS, Weitzman E. 1995. The effects of focused stimulation for promoting vocabulary in young children with delays: A pilot study. Journal of Children's Communication Development, *17*(2), pp.39-49. https://doi.org/10.1177/152574019501700205

33)Romeo, R.R., Leonard, J.A., Robinson, S.T., West, M.R., Mackey, A.P., Rowe, M.L. and Gabrieli, J.D., 2018. Beyond the 30-million-word gap: Children's conversational exposure is associated with language-related brain function. https://doi.org/10.1177/0956797617742725

34)Wasik, B. A., & Jacobi-Vessels, J. L. (2017). Word play: Scaffolding language development through child-directed play. *Early Childhood Education Journal, 45*(6), pp. 769-776. https://doi.org/10.1007/s10643-016-0827-5

For peer review only

35)Zimmerman, I. L., Steiner, V. G., & Pond, R. E. (2002). *Preschool Language Scale, Fourth Edition (PLS-4)*.https://doi.org/10.1037/t15140-000

36)McLeod, S., Harrison, L.J. and McCormack, J., 2012. The intelligibility in context scale: Validity and reliability of a subjective rating measure. https://doi.org/10.1044/1092-4388(2011/10-0130)

37)Sugden, E., Baker, E., Williams, A.L., Munro, N. and Trivette, C.M., 2020. Evaluation of parent-and speech-language pathologist–delivered multiple oppositions intervention for children with phonological impairment: A multiple-baseline design study. https://doi.org/10.1044/2019_AJSLP-18-0248

38)Curran, M. & Owen Van Horne, A., 2019. Use of Recast Intervention to Teach Causal Adverbials to Young Children With Developmental Language Disorder Within a Science Curriculum: A Single Case Design Study. https://doi.org/10.1044/2018_AJSLP-17-0164

39)Wren, Y., Harding, S., Goldbart, J. and Roulstone, S., 2018. A systematic review and classification of interventions for speech-sound disorder in preschool children. https://doi.org/10.1111/1460-6984.12371

40)Frizelle, P., Tolonen, A.K., Tulip, J., Murphy, C.A., Saldana, D. and McKean, C., 2021. The influence of quantitative intervention dosage on oral language outcomes for children with developmental language disorder: A systematic review and narrative synthesis. https://doi.org/10.1044/2020_LSHSS-20-00058

41)Carson, L., Baker, E. and Munro, N., 2022. A Systematic Review of Interventions for Late Talkers: Intervention Approaches, Elements, and Vocabulary Outcomes. https://doi.org/10.1044/2022_AJSLP-21-00168

42)Murray, E., Power, E., Togher, L., McCabe, P., Munro, N. and Smith, K., 2013. The reliability of methodological ratings for speechBITE using the PEDro-P scale. https://doi.org/10.1111/1460-6984.12007

43)Tate, R.L., Perdices, M., Rosenkoetter, U., Wakim, D., Godbee, K., Togher, L. and McDonald, S., 2013. Revision of a method quality rating scale for single-case experimental designs and n-of-1 trials: The 15-item Risk of Bias in N-of-1 Trials (RoBiNT) Scale. https://doi.org/10.1080/09602011.2013.824383

44)Law, J., Charlton, J., Dockrell, J., Gascoigne, M., McKean, C. and Theakston, A., 2017. Early Language Development: Needs, provision and intervention for pre-school children from socio-economically disadvantaged backgrounds. London: Education Endowment Foundation. https://educationendowmentfoundation.org.uk/public/files/Law_et_al_Early_Language_Development_final.pdf

45)Hoffmann, T.C., Glasziou, P.P., Boutron, I., Milne, R., Perera, R., Moher, D., Altman, D.G., Barbour, V., Macdonald, H., Johnston, M. and Lamb, S.E., 2014. Better reporting of

interventions: template for intervention description and replication (TIDieR) checklist and guide. https://doi.org/10.1136/bmj.g1687

46)Warren, S.F., Fey, M.E. & Yoder, P.J. 2007. Differential treatment intensity research: A missing link to creating optimally effective communication interventions. https://doi.org/10.1002/mrdd.20139

47)Campbell, M., McKenzie, J.E., Sowden, A., Katikireddi, S.V., Brennan, S.E., Ellis, S., Hartmann-Boyce, J., Ryan, R., Shepperd, S., Thomas, J. and Welch, V., 2020. Synthesis without meta-analysis (SWiM) in systematic reviews: reporting guideline. https://doi.org/10.1136/bmj.l6890

48)Morris C, Simkiss D, Busk M*, et al.*Setting research priorities to improve the health of children and young people with neurodisability: a British Academy of Childhood Disability-James Lind Alliance Research Priority Setting Partnership. doi: 10.1136/bmjopen-2014-006233

49)Rodgers, L., and Harford., D. Collaborating with parents to identify outcomes for a preschool speech and language intervention. ESLA Congress 2022: New frontiers in speech and language therapy: advancing practice, research and education. DOI:10.13140/RG.2.2.34626.45763 https://www.researchgate.net/publication/362910019_Collaborating_with_parents_to_identify_outcomes_for_a_pre-school_speech_and_language_intervention

50)Rodgers, L., and Harford., D. 2022. Part of the family. RCSLT Bulletin. Issue 832, autumn 2022.

51)Staniszewska, S., Brett, J., Simera, I., Seers, K., Mockford, C., Goodlad, S., Altman, D.G., Moher, D., Barber, R., Denegri, S. and Entwistle, A., 2017. GRIPP2 reporting checklists: tools to improve reporting of patient and public involvement in research. https://doi.org/10.1136/bmj.j3453

For peer review only

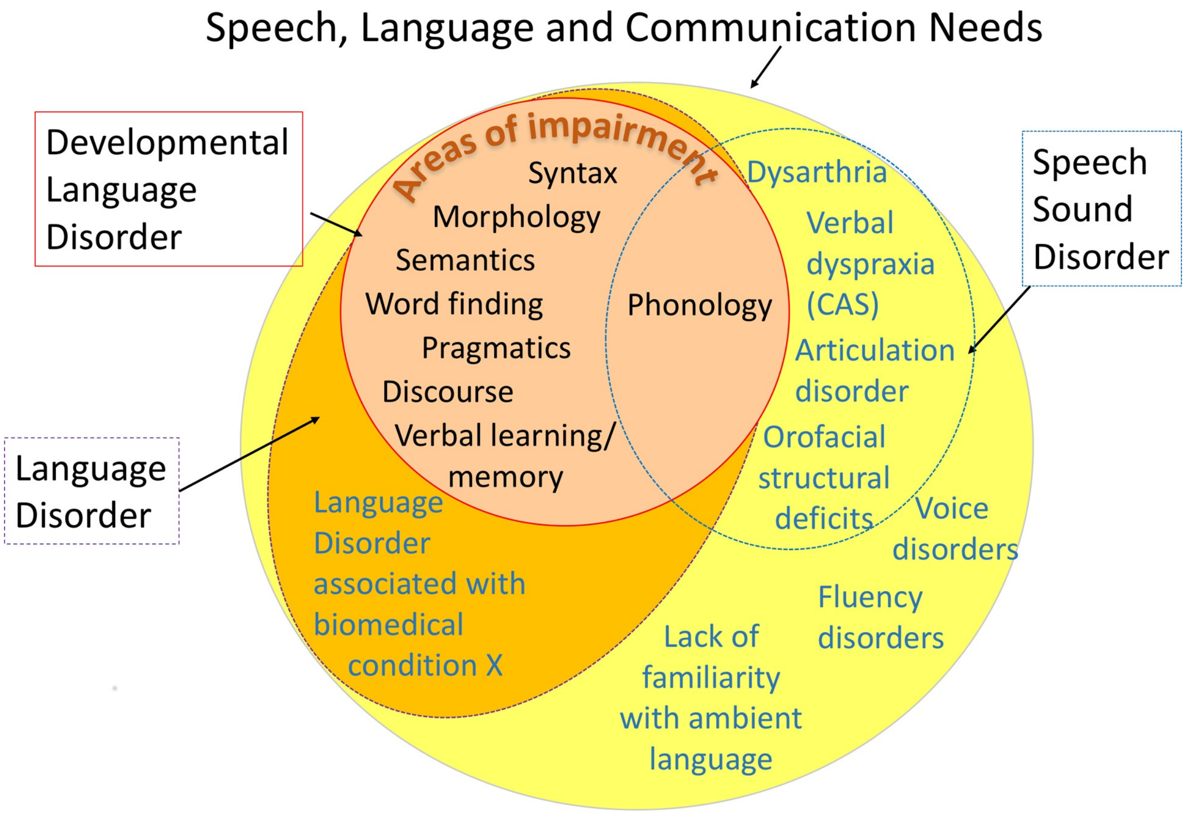
Speech, Language and Communication Needs
Developmental Language Disorder
Language Disorder
Speech Sound Disorder
Areas of impairment
Syntax
Morphology
Semantics
Word finding
Pragmatics
Discourse
Verbal learning/ memory
Phonology
Language Disorder associated with biomedical condition X
Dysarthria
Verbal dyspraxia (CAS)
Articulation disorder
Orofacial structural deficits
Voice disorders
Fluency disorders
Lack of familiarity with ambient language

SUPPLEMENTARY MATERIALS 1:
SEARCH STRATEGY FOR EACH DATABASE

1.EBSCO *(Medline, APA Psychinfo, CINAHL, Communication Source)*

Paediatric **OR** paediatrics **OR** children **OR** child **OR** infant **OR** infants **OR** schoolchild **OR** schoolchildren **OR** preschool **OR** "early years" **OR** kindergarten *(AB)*
NOT
Teenage **OR** teenagers **OR** adolescent **OR** adolescents *(SU)*

AND

Therapy **OR** intervention **OR** interventions **OR** treatment **OR** treatments **OR** programme **OR** programmes **OR** program **OR** programs **OR** teaching **OR** instruction **OR** approach **OR** approaches **OR** technique **OR** techniques **OR** strategy **OR** strategies **OR** activity **OR** activities **OR** class **OR** classes *(AB)*

AND

"language delay" **OR** "language disorder" **OR** "specific language impairment" **OR** "language impairment" **OR** "language difficulties" **OR** "developmental language disorder" **OR** "late talker" **OR** "speech delay" **OR** "speech disorder" **OR** "speech sound disorder" **OR** "speech intelligibility" **OR** "intelligible speech" **OR** "speech comprehensibility" **OR** "comprehensible speech" *(AB)*
NOT
"sign language" **OR** "mental retardation" **OR** autism **OR** "autistic spectrum disorder" **OR** Asperger **OR** "cleft lip" **OR** "cleft palate" **OR** deaf **OR** "cerebral palsy" **OR** aphonia **OR** geriatrics **OR** "down syndrome" **OR** "cochlear implant" **OR** "autistic spectrum disorder" **OR** "autistic spectrum disorders" **OR** "autism disorder" **OR** "autistic disorder" (*SU)*

Limiters: 2012-current

2.Ovid *(Emcare)*
Paediatric **OR** paediatrics **OR** children **OR** child **OR** infant **OR** infants **OR** schoolchild **OR** schoolchildren **OR** preschool **OR** "early years" **OR** kindergarten *(AB)*
NOT
Teenage **OR** teenagers **OR** adolescent **OR** adolescents *(SH)*

AND

Therapy **OR** intervention **OR** interventions **OR** treatment **OR** treatments **OR** programme **OR** programmes **OR** program **OR** programs **OR** teaching **OR** instruction **OR** approach **OR** approaches **OR** technique **OR** techniques **OR** strategy **OR** strategies **OR** activity **OR** activities **OR** class **OR** classes *(AB)*

For peer review only

AND

“language delay” **OR** “language disorder” **OR** “specific language impairment” **OR** “language impairment” **OR** “language difficulties” **OR** “developmental language disorder” **OR** “late talker” **OR** “speech delay” **OR** “speech disorder” **OR** “speech sound disorder” **OR** “speech intelligibility” **OR** “intelligible speech” **OR** “speech comprehensibility” **OR** “comprehensible speech” *(AB)*

NOT

“sign language” **OR** “mental retardation” **OR** autism **OR** “autistic spectrum disorder” **OR** Asperger **OR** “cleft lip” **OR** “cleft palate” **OR** deaf **OR** “cerebral palsy” **OR** aphonia **OR** geriatrics **OR** “down syndrome” **OR** “cochlear implant” **OR** “autistic spectrum disorder” **OR** “autistic spectrum disorders” **OR** “autism disorder” **OR** “autistic disorder” (*SH*)

Limiters: 2012-current

3.ERIC (descriptor terms)

"speech impairments" **OR** "language impairments" **OR** intelligibility **OR** "expressive language" **AND** "speech language pathology" **OR** "speech therapy" **AND** "young child" **OR** "preschool children" **OR** toddlers **OR** "early childhood education”

Limiters: peer reviewed only
Pre-2012 studies to be removed manually following the search, within Refworks

For peer review only

SUPPLEMENTARY MATERIALS 2:
EXCLUSION SCREENING GUIDANCE

Population

Inclusion

80% children aged 2:0 and 5:11; idiopathic phonological speech production difficulties and/or difficulties relating to oral vocabulary; difficulties identified on standardised assessments, parental/and or professional observation reports, and/or pre-intervention baseline probes; idiopathic speech/language needs

Exclusion

Children with typically developing speech/language skills; speech/language difficulties not caused by or associated with a condition with a known impact on communication e.g. autism, deafness, cerebral palsy, cleft lip/palate, dysarthria

Title/abstract level specific: still include if any of the exclusion criteria remains unknown and the inclusion criteria is met or unknown.

Intervention

Inclusion: Any setting, deliverer, mode of delivery

Exclusion: N/A

Comparator

Inclusion: Empirical evaluation of intervention effectiveness from RCTs, experimental and quasi-experimental studies, case studies/within groups designs.

Exclusion: Assessment at a single timepoint pre and post intervention, with no comparator.

Title/abstract level specific: still include if any of the exclusion criteria remains unknown and the inclusion criteria is met or unknown.

Outcome

Inclusion: Measure of oral vocabulary and/or speech comprehensibility. Include intelligibility measures as a proxy for comprehensibility. Include proximal measures of vocabulary development that may arise from syntactic assessments, such as the number of different words (NDW). Outcomes must be evaluated via standardised assessment, probes, and/or observational ratings or scales. Post intervention assessment at any timepoint.

Exclusion: Composite measures where individual results for speech comprehensibility/intelligibility and/or oral vocabulary are not completed in a separate analysis. Purely syntactic measures of change, such as the mean length of utterance in morphemes (MLUm).

Title/abstract level specific: still include if any of the exclusion criteria remains unknown and the inclusion criteria is met or unknown. Still include if primary outcomes do not relate to

For peer review only

vocabulary/speech comprehensibility, but where the inclusion criteria are either met or unknown.

For peer review only

SUPPLEMENTARY MATERIALS 3:
DRAFT CONTENT FOR THE DATA EXTRACTION FORM

1.General information/study details

- Date
- Title
- Location (country)
- Language the intervention was in
- Study design
- Comparator
- No. participants (incl. Participants in control/alternative experimental group if relevant)
- Why- goals/aims of the overall intervention
- Specific outcome/s measured of relevance to this review
- Is this a primary outcome of the intervention?
- Do the authors refer to a protocol which was made available before recruitment commenced?
- If a pre-study protocol exists, do the outcomes and results section of the published report align with this protocol?

2.Population characteristics

- Age
- Male/female
- Languages spoken
- Ethnicity
- SES details as reported (e.g. parental education/employment)
- Assessments used to identify DLD/SSD features
- Pre-intervention speech assessment levels
- Pre-intervention language assessment levels (comprehension as well as expressive)
- Phonological SSD sub type- was this specified?
- If phonological SSD sub type was not specified, was it indicated-and if so, how? *(e.g. through the selection of treatment targets, baseline assessment results)*

3.Intervention characteristics

- Setting (e.g. home, nursery/school, clinic)
- Was the intervention modified at any point? If so, how?
- Techniques within the intervention
- Rationale for each technique/the technique within the wider approach
- Mode of delivery of the technique (e.g. fully face to face in clinic; hybrid in clinic with some carryover over home and/or nursery; virtual delivery)
- Was technique delivery implicit (e.g. listening to an adults' model) or explicit (e.g. being asked to repeat)?
- The wider activity/game the technique is part of (e.g. shared book reading, child led play, everyday routines, a combination of these)

For peer review only

- Dose frequency of the individual technique (no. times delivered per session, day, across a week)
- Dose frequency of the intervention as a whole
- Total duration of the technique (the time period between which the technique is used)
- Total duration of the intervention (the time period/duration of the intervention as a whole)
- Additional dosage information as reported
- Deliverer/s of the technique
- If not SLT, how the person was trained to deliver the technique
- Materials used to carry out the technique
- Was intervention fidelity measured? Report on this if so

4.Outcomes

- Summary of exact outcome *(e.g. comprehensibility with parents or with teaching staff? Spontaneous word production in free play or picture naming?)*
- Measure/s used
- Timepoints
- Reported effect
- Direction of effect (benefit vs no benefit/harm)

For peer review only

PRISMA-P (Preferred Reporting Items for Systematic review and Meta-Analysis Protocols) 2015 checklist: recommended items to address in a systematic review protocol*

Section and topic	Item No	Checklist item	Location
**ADMINISTRATIVE INFORMATION**			
Title:			
Identification	1a	Identify the report as a protocol of a systematic review	Page 1 (title page)
Update	1b	If the protocol is for an update of a previous systematic review, identify as such	Page 2 (abstract) further details on page 5 (broader context: an intervention development study)
Registration	2	If registered, provide the name of the registry (such as PROSPERO) and registration number	Page 2 (abstract), page 6
Authors:			
Contact	3a	Provide name, institutional affiliation, e-mail address of all protocol authors; provide physical mailing address of corresponding author	Page 1 (title page)
Contributions	3b	Describe contributions of protocol authors and identify the guarantor of the review	Pages 13/14
Amendments	4	If the protocol represents an amendment of a previously completed or published protocol, identify as such and list changes; otherwise, state plan for documenting important protocol amendments	Page 2 (abstract); further details on page 9 (search strategy)
Support:			
Sources	5a	Indicate sources of financial or other support for the review	Page 14
Sponsor	5b	Provide name for the review funder and/or sponsor	Page 14
Role of sponsor or funder	5c	Describe roles of funder(s), sponsor(s), and/or institution(s), if any, in developing the protocol	Page 14
**INTRODUCTION**			
Rationale	6	Describe the rationale for the review in the context of what is already known	Pages 3-6 (introduction)
Objectives	7	Provide an explicit statement of the question(s) the review will address with reference to participants, interventions, comparators, and outcomes (PICO)	Page 6 (objectives and research questions) further details on pages 7/8 (eligibility criteria)
**METHODS**			
Eligibility criteria	8	Specify the study characteristics (such as PICO, study design, setting, time frame) and report characteristics (such as years considered, language, publication status) to be used as criteria for eligibility for the review	Page 6 (objectives and research questions); further details on

For peer review only

			pages 7/8 (eligibility criteria)
Information sources	9	Describe all intended information sources (such as electronic databases, contact with study authors, trial registers or other grey literature sources) with planned dates of coverage	Pages 8-9 (information sources)
Search strategy	10	Present draft of search strategy to be used for at least one electronic database, including planned limits, such that it could be repeated	Supplementary materials document 1
Study records:			
Data management	11a	Describe the mechanism(s) that will be used to manage records and data throughout the review	Pages 9/10 (study records, selection and data collection process)
Selection process	11b	State the process that will be used for selecting studies (such as two independent reviewers) through each phase of the review (that is, screening, eligibility and inclusion in meta-analysis)	Pages 9/10 (study records, selection and data collection process)
Data collection process	11c	Describe planned method of extracting data from reports (such as piloting forms, done independently, in duplicate), any processes for obtaining and confirming data from investigators	Page 10 (study records, selection and data collection process); page 11 (data items); supplementary materials document 3
Data items	12	List and define all variables for which data will be sought (such as PICO items, funding sources), any pre-planned data assumptions and simplifications	Page 11(data items) supplementary materials document 3
Outcomes and prioritization	13	List and define all outcomes for which data will be sought, including prioritization of main and additional outcomes, with rationale	Page 11 (outcomes and prioritisation)
Risk of bias in individual studies	14	Describe anticipated methods for assessing risk of bias of individual studies, including whether this will be done at the outcome or study level, or both; state how this information will be used in data synthesis	Page 10 (risk of bias/internal validity); page 11 (risk of bias in individual studies); page 11 (quantitative data)
Data synthesis	15a	Describe criteria under which study data will be quantitatively synthesised	Page 11 (data synthesis: quantitative data)
	15b	If data are appropriate for quantitative synthesis, describe planned summary measures, methods of handling data and methods of combining data from studies, including any planned exploration of consistency (such as I^2, Kendall's τ)	Not applicable
	15c	Describe any proposed additional analyses (such as sensitivity or subgroup analyses, meta-regression)	Not applicable

For peer review only

	15d	If quantitative synthesis is not appropriate, describe the type of summary planned	Page 12 (data synthesis: qualitative data)
Meta-bias(es)	16	Specify any planned assessment of meta-bias(es) (such as publication bias across studies, selective reporting within studies)	Page 13 (meta-bias(es))
Confidence in cumulative evidence	17	Describe how the strength of the body of evidence will be assessed (such as GRADE)	Page 13 (strength in body of evidence)

*** It is strongly recommended that this checklist be read in conjunction with the PRISMA-P Explanation and Elaboration (cite when available) for important clarification on the items. Amendments to a review protocol should be tracked and dated. The copyright for PRISMA-P (including checklist) is held by the PRISMA-P Group and is distributed under a Creative Commons Attribution Licence 4.0.**

From: Shamseer L, Moher D, Clarke M, Ghersi D, Liberati A, Petticrew M, Shekelle P, Stewart L, PRISMA-P Group. Preferred reporting items for systematic review and meta-analysis protocols (PRISMA-P) 2015: elaboration and explanation. BMJ. 2015 Jan 2;349(jan02 1):g7647.

For peer review only

BMJ Open

The shared characteristics of intervention techniques for oral vocabulary and speech comprehensibility in pre-school children with co-occurring features of developmental language disorder and a phonological speech sound disorder: protocol for a systematic review with narrative synthesis

Journal:	*BMJ Open*
Manuscript ID	bmjopen-2022-071262.R2
Article Type:	Protocol
Date Submitted by the Author:	05-May-2023
Complete List of Authors:	Rodgers, Lucy; City University of London, Language and Communication Science; Sussex Community NHS Foundation Trust, Children's Speech and Language Therapy<br>Botting, Nicola ; City University of London, Department of Language and Communication Science<br>Cartwright, Martin; City University of London, Department of Health Services Research and Management<br>Harding, Sam; North Bristol NHS Trust, Bristol Speech and Language Therapy Research Unit<br>Herman, Rosalind; City University of London, Department of Language and Communication Science
**Primary Subject Heading**:	Paediatrics
Secondary Subject Heading:	Communication
Keywords:	Community child health < PAEDIATRICS, Speech pathology < OTOLARYNGOLOGY, Developmental neurology & neurodisability < PAEDIATRICS

SCHOLARONE™ Manuscripts

The shared characteristics of intervention techniques for oral vocabulary and speech comprehensibility in pre-school children with co-occurring features of developmental language disorder and a phonological speech sound disorder: protocol for a systematic review with narrative synthesis

AUTHORS:
Lucy Rodgers. Department of Language and Communication Science, Myddelton Street Building, City University, London, UK. https://orcid.org/0000-0001-6585-7206

Prof. Nicola Botting. Department of Language and Communication Science, City University, London, UK.

Dr Martin Cartwright. Department of Health Services Research and Management, City University, London, UK.

Dr Sam Harding. Bristol Speech and Language Therapy Research Unit, North Bristol NHS Trust, Bristol, UK. https://orcid.org/0000-0002-5870-2094

Prof. Rosalind Herman. Department of Language and Communication Science, City University, London, UK. http://orcid.org/0000-0001-5732-9999

Correspondence to:
Lucy Rodgers
Department of Language and Communication Science, Myddelton Street Building, City University of London, London EC1V 0HB, UK
Lucy.Rodgers@city.ac.uk

Word count: 4,748

Keywords: Language Development Disorders; Speech Sound Disorder; Child Language; Speech-Language Pathology

For peer review only

ABSTRACT

Introduction

Evidence suggests that over a third of young children with Developmental Language Disorder (DLD) or Speech Sound Disorder (SSD) have co-occurring features of both. A co-occurring DLD and SSD profile is associated with negative long-term outcomes relating to communication, literacy and emotional wellbeing. However, the best treatment approach for young children with this profile is not understood. The aim of the proposed review is to identify intervention techniques for both DLD and SSD, along with their shared characteristics. The findings will then be analysed in the context of relevant theory. This will inform the content for a new or adapted intervention for these children.

Methods and analysis

This search will build on a previous systematic review by Roulstone et al. (2015) but with a specific focus on oral vocabulary (DLD outcome) and speech comprehensibility (SSD outcome). These outcomes were identified by parents and Speech and Language Therapists within the pre-study stakeholder engagement work. The following databases will be searched for articles from January 2012 onwards: Ovid EMCare, Medline Complete, CINAHL, APA PsychINFO, Communication Source and ERIC. Two reviewers will independently perform the title/abstract screening and the full text screening with the exclusion criteria document being revised in an iterative process. Articles written in languages other than English will be excluded. Data will be extracted regarding key participant and intervention criteria, including technique dosage and delivery details. This information will then be pooled into a structured narrative synthesis.

Ethics and dissemination

Ethical approval is not needed for a systematic review protocol. Dissemination of findings will be through peer-reviewed publications, social media, and project steering group networks.

Study registration number: PROSPERO, CRD4202237393.

For peer review only

Strengths and limitations of this study

- This protocol follows the referred Reporting Items for Systematic Review and Meta-Analysis Protocols (PRISMA-P) guidelines.
- Electronic databases spanning medicine, education, and psychology will be searched.
- Electronic databases in languages other than English will not be searched.
- Meta-bias(es) within the literature cannot be fully controlled.
- The level of detail within intervention reporting, as per the TIDieR guidelines, has the potential to vary amongst studies.

INTRODUCTION

Within the field of child language disorders, there are often overlapping or co-occurring difficulties which create unique patient experiences. Yet, while there is ample literature on treatment for singly occurring difficulties, there is a notable gap in evidence for treating children with co-occurring disorders. This review focusses on intervention for children who have co-occurring features of both Developmental Language Disorder (DLD) and Speech Sound Disorder (SSD).

Co-occurring Developmental Language Disorder (DLD) and Speech Sound Disorder (SSD)
An estimated 7.58% of 4 year olds present with features of a Developmental Language Disorder (DLD)(1). DLD is characterised by idiopathic difficulties in using and understanding spoken language (2). One feature is limited vocabulary development (2), which has a known association with childhood temper tantrums/mental health, and later language and literacy skills (3,4). Such features of DLD may co-occur with a Speech Sound Disorder (SSD); that is, difficulties in producing speech sounds (5). An estimated 3.4% of 4- year olds have SSD (6). One of the most devastating impacts SSD is the impact on a child's ability to make themselves understood to others in everyday life (7). The term for this is speech comprehensibility (8). A related term, speech intelligibility, refers to the acoustic-phonetic decoding of utterances, and is very closely related to speech comprehensibility as both are linked to the functional use of speech. As with limited vocabulary, poor speech comprehensibility/intelligibility within the early years have also been associated with negative longer-term outcomes, including persisting speech difficulties (9) and poor literacy skills (10,11). Although it is typical for very young children not to be fully understood to those around them as their speech develops, by 4 years of age a child would typically be at least 50% intelligible (12).

Thirty six percent of 4-year olds with idiopathic SSD also have oral (i.e. expressive-spoken) language features of DLD (6). This high rate of co-occurrence is in keeping with historical research in the area (13), as well as study data from clinical caseloads (14). The combined impact of co-occurring features of DLD/SSD is twofold; for example, for a child with limited oral vocabulary and speech comprehensibility, not only are they unable to use many words, but the limited words they do have will not be understood to others within their daily lives. It therefore may be unsurprising that co-occurring phonological DLD/SSD features in early childhood are associated with negative long-term outcomes relating to literacy (15,16) and communication (17,18), with downstream consequences for quality of life (18,19) and emotional wellbeing (20). Consequently, access to effective and appropriately targeted intervention for children with this profile is crucial.

Phonological SSDs are the most frequently presenting SSD subtype (5), and occur when a child has difficulties with manipulating the different sound contrasts (phonemes) which are needed to form words (21). There are different types of phonological SSDs, including consistent phonological disorder (where the child makes consistent sound omissions or substitutions) and inconsistent phonological disorder (where these errors have no consistent pattern) (21). Research highlights a known link between DLD and phonological SSDs, as both disorders are underpinned by shared linguistic deficits (2). This overlap is represented in the seminal CATALISE DLD consensus paper (2). In contrast to phonological

For peer review only - http://bmjopen.bmj.com/site/about/guidelines.xhtml

SSDs, the CATALISE authors' speech, language and communication needs diagram highlights how other SSD subtypes, such as motor based SSDs like dysarthria, have a less marked overlap with DLD. Although non phonological SSDs such as articulation disorder and childhood dyspraxia of speech (CAS) could also be idiopathic, other non phonological SSDs often are not. Due to their significant overlap with DLD which has no known causation, this review will focus on phonological SSDs which are also idiopathic in nature.

Speech, language and communication needs are illustrated in Figure 1 (2).

The overlap between language and phonological SSDs is further supported by studies on the speech and language development of young children, where complex and bi-directional relationships between the development of individual sounds (phonology) and words (the lexicon) have been identified (22,23). For example, the first words of young children primarily consist of the speech sounds already established within their emerging phonological inventory (23). This relationship between phonology and the lexicon may have implications for intervention with children with co-occurring features of DLD and a phonological SSD. For example, growth in vocabulary and/or the strengthening of phonological representations has the potential to impact speech and vocabulary development concurrently through a process known as 'lexical restructuring' (24). A further psycholinguistic theory of potential relevance is the speech processing model (25), which suggests that individual children with co-occurring features of DLD/a phonological SSD may have difficulties at one or more levels of speech processing, rather than just with phonological representations alone. Such theories are important within interventions for co-occurring DLD/phonological SSD as they can be used to inform intervention content and delivery.

Current interventions for pre-school co-occurring DLD/SSD

Although this overlap exists between DLD and phonological SSDs, there is currently a paucity of theoretically informed interventions which have been specifically developed for this group (26). Additionally, intervention studies within existence primarily target morphosyntactic aspects of expressive language, alongside accuracy of speech sound production (26,27). However, for younger children with this profile, and children whose features of DLD are more severe, building vocabulary is typically targeted in speech and language therapy prior to morphosyntax (28).

'Child Talk' (29) was a large National Institute of Health Research (NIHR) funded mixed methods programme of work, including a systematic review. This involved investigating the use of early years' speech and language therapy interventions. The findings led to the specification of a) a typology of early years' speech and language therapy (SLT) intervention, b) key intervention ingredients for each typology theme. A technique can be described as "the specific teaching behaviours/actions thought to effect change" (30). The findings highlighted that for children with co-occurring features of DLD/SSD, clinicians often adapt existing interventions by selecting and combining different techniques. This enables them to use their knowledge and experience to provide the best treatment that they can (29,31).

For peer review only

Although our knowledge of what techniques work best for children with this profile is limited, techniques identified might be related to underlying theories of potential relevance. For example, language modelling is typically linked to growth in expressive language (32). However, based on the lexical restructuring hypothesis, it is hypothesised that the subsequent impact of this language growth on the accuracy and segmentation of the child's phonological representations could also influence their phonological speech sound production (24). Using various techniques to 'build things into play' was also highlighted in Child Talk. Theoretically, this is supported by the latest research on the brain basis of speech and language learning, which indicates that learning best takes place within interactions which are meaningful for the child (33,34). Romeo et al. (2018) found that Broca's area of the brain became activated in response to a child being exposed to meaningful back and forth interactions, rather than in response to passively 'hearing' words. Considering this, it is hypothesised that this technique supports speech and language learning through capitalising on the child's heightened attention and motivation during the play activity.

These considerations highlight a valuable opportunity for an intervention specific to this clinical group to be developed, utilising techniques which can be supported by relevant theory. Due to the current paucity of evidence, the associated negative impact of this co-occurring profile on long term outcomes, and the high level of presentation on clinical caseloads, there is an urgent need for such intervention development to take place. The first stage in this development would be to conduct a systematic review to identify potential techniques of relevance.

Broader context: an intervention development study

The proposed review updates the systematic review findings from 'Child Talk' (29) whilst refining the focus to techniques within interventions for children with features of DLD or a phonological SSD. Techniques will be extracted from included studies and then analysed in relation to shared characteristics and underpinning theory. The synthesis will then be used to inform the content of a new intervention which is being developed for young children with co-occurring features of DLD/phonological SSD.

Both DLD and SSD are heterogenous disorders (2,21), and therefore have a range of associated outcomes. This review, and body of intervention development work it is a part of, will focus exclusively on the outcomes of oral vocabulary (DLD outcome) and speech comprehensibility (SSD outcome). This is due to the afore mentioned impact of such difficulties on the everyday lives of young children; this decision is also elaborated on in the 'patient and public involvement' section of this paper.

Based on the dose form framework (30), shared characteristics for DLD/phonological SSD intervention techniques may include similarities in:

1) *who* delivers the technique; for example, is it the parent, clinician, or both?
2) *where* the technique is delivered; for example, at home, nursery, clinic or a combination of these?
3) the nature of *technique delivery*; for example, is the activity presented in an adult led structured game, play, everyday routines, or a combination of these?

For peer review only - http://bmjopen.bmj.com/site/about/guidelines.xhtml

Underpinning theory may relate to:

1)The lexical restructuring hypothesis (24)
2) Psycholinguistic models of speech and language development; such as the speech processing model (25)
3)The neural basis for speech and language development; for example, the role of meaningful interactions within language learning (33)

Objectives

The overarching aim of the review is to bring together intervention techniques from DLD and phonological SSD interventions. The objectives within this are to:

1. Identify the shared core characteristics of the techniques; this includes the deliverer, place of delivery, format of delivery and nature of delivery *(e.g. child or adult led)*
2. Compare and synthesise the shared core characteristics of the techniques in relation to underlying theory
3. Establish the best available evidence for interventions that incorporate these core characteristics of the intervention techniques

Research questions

1. What are the shared core characteristics of intervention techniques in preschool interventions targeting speech comprehensibility and/or oral vocabulary?
2. How do these shared core characteristics relate to underlying theory?
3. What evidence is there for the effect of interventions that incorporate these core characteristics of intervention techniques?

METHODS AND ANALYSIS

Study registration

In accordance with the guidelines, our systematic review protocol has been registered with the International Register of Systematic Reviews (PROSPERO) on 16/12/2022 (registration number CRD42022373931). In the event of any amendments to methodology set out below, the date of each amendment will be accompanied by a description of the change and the rationale in either the Prospero register and/or the final results paper.

Eligibility criteria

The eligibility criteria stated below are in line with the criteria from the original 'Child Talk' systematic review (29) with amendments according to the objectives of the current review. Most importantly, this review will focus specifically on the 'expressive language' and 'speech' themes generated from their initial typology of early years' Speech and Language Therapy (SLT) interventions, as these themes encompass the two outcomes for which we are seeking to identify techniques.

For peer review only

Study designs

Included studies must report on an empirical evaluation of the effectiveness of an intervention. To ensure we identify all relevant literature, a range of study designs will be included. These include randomised control trials (RCTs), experimental and quasi-experimental studies, within subjects designs (e.g. pre-post studies) and case studies (which may include multiple baseline or other systematic manipulation of the intervention). Studies which report on single timepoint (e.g. cross-sectional studies) will be excluded. Studies focusing on efficacy, including lab-based training, will not be excluded if all other inclusion criteria are met. This is because information on the efficacy of speech/language learning techniques can be gleaned from these studies, although careful consideration will be given to how these results are integrated into the narrative analysis (further information on this is provided under 'data synthesis').

Population

To capture the age group most typically seen within clinical services, 80% of children within included studies must have been aged between 2:0 and 5:11 years. Additionally, although this review is part of a wider intervention development study for children aged 3 and 4 years, an expanded age range within this review will help to ensure all that techniques of potential relevance will be captured. The children within included studies must have presented with phonological speech production difficulties and/or difficulties relating to oral vocabulary, with all subtypes of phonological SSD included (e.g. consistent and inconsistent phonological disorder, phonological delay).These difficulties may be identified by standardised assessments such as the Preschool Language Scale (35), parental and/or professional observation reports such as the intelligibility in context scale (36) and/or probes. Probes may also be used to assess progress through the repeated measurement of the dependent variable before, during and after the intervention. As already observed in the literature, common probes within speech and language therapy interventions may include a selection of words containing the child's targeted speech sound/s or vocabulary (37,38). In keeping with the afore mentioned diagnostic description within CATALISE (2), included papers must state that the participants' needs had no obvious cause, i.e., excluding children with neurodevelopmental differences that have a known association with speech and/or language development, such as Autism or Cerebral Palsy. Due to the challenges in diagnosing DLD in very young children (2), and in order to maximise the identification of potentially relevant intervention techniques, studies will be included where the child does not have a formal diagnosis of DLD but is a late talker.

Interventions

We will include studies reporting on interventions delivered in any setting (e.g., home based, clinic) or format (e.g., face to face, online). The deliverer may be a speech and language therapist, speech and language therapy assistant, or equivalent professional (including education staff), and the intervention may involve professionals training up others (e.g., parents) to deliver some or all of the intervention.

Comparators

For peer review only

Comparators for included studies may be a control without an intervention (including multiple baseline and within subjects designs) or an alternative experimental group (i.e., intervention comparison).

Outcomes
Included papers must measure the effectiveness of the intervention on a) oral vocabulary, and/or b) speech comprehensibility. These outcomes must be evaluated via standardised assessment, probes, and/or observational ratings or scales.

If composite speech and language assessments are used, studies must report on the separate sub test results for oral vocabulary and/or speech comprehensibility to be included.

Studies with only syntactic measures of language change will be excluded; this includes mean length of utterance in morphemes (MLUm). However, they will be included if a proximal measure of vocabulary change is used alongside syntactic measures, such as the number of different words (NDW). Other outcome measures related to oral vocabulary might inlcude parent report instruments and type-token ratios from language samples.

Speech comprehensibility is the SSD outcome in focus. As previously mentioned, comprehensibility and intelligibility are overlapping but differing constructs, with a shared focus on functional human communication (8). Therefore, we will also include studies with an outcome of improved speech intelligibility as a proxy for comprehensibility. This was deemed more suitable than using measures such as PCC as a proxy for comprehensibility, with their focus being on speech accuracy. Due to the very recent consensus in terminology, measures for comprehensibility might include measures with ‘intelligibility’ within their title, such as the ‘Intelligibility in Context’ Scale (ICS), which is becoming increasingly utilised in SSD intervention research (36,38). In the ICS, parents are asked to rate their child’s speech comprehensability according to the communication partner they are with within their everyday envionments, thus providing high ecological validity. We will also include non-parent/significant rated measures when looking at comprehensibility. For example, orthography based approaches where raters are not known to the child (39). Given the recent clarification on consensus on intelligibility vs comprehensibility (8), it could be argued that this approach falls between the two, with there being less focus on who the speaker’s communication partner is, and the wider context which the interaction takes place in (8). Regardless, such studies will still be included, as the results still relate to functional human communication.

Information sources
The search will be conducted in Ovid EMCare, Medline Complete, CINAHL, APA PsychINFO, ERIC and Communication Source. These sources have been selected as they encompass the fields of health (medical, nursing and allied health professions), speech and language therapy, education and psychology and have been successfully utilised in previous reviews in the field (26,40). To support with literature saturation, supplementary search methods will be employed; this includes screening the reference lists from prominent reviews in the field post 2012 (41,42). We have selected reviews from 2012 onwards due to the original search going up to this date (29). Reference lists from included papers within the current

For peer review only - http://bmjopen.bmj.com/site/about/guidelines.xhtml

search will also be screened for potential study eligibility. Forward citation searches in Web of Science (using the core collection) will also be carried out, with additional searches in Scopus if the titles are not available in Web of Science.

Due to resource constraints, articles written in languages other than English will be excluded. However, articles written in English where the participants speak languages other than English will be included. Additionally, grey literature searching will be confined to the inclusion of theses/dissertations, via the databases stated above. Thesis/dissertations have been selected as although the original review (29) included a range of grey literature, thesis/dissertations were the only grey literature sources which contributed studies within the final included papers. In keeping with the original review, thesis/dissertations will only be included when a corresponding journal article cannot be found for the study.

Search strategy

Together with support from a specialist librarian, we will conduct an update of the original 'Child Talk' systematic review (29), searching articles from January 2012 to the present day. One of the researchers (SH) undertaking the current search also led on the original review. Relevant studies from the original 'speech' and 'expressive language' typology themes within the original 'Child Talk' review have already been located by reviewing the recorded outcomes for each study as stated on the original data extraction spreadsheet. Out of 41 papers from the 'speech' theme, two were found to address the outcome of comprehensibility/intelligibility. From the 30 papers within the 'expressive language' theme, 12 were found to include oral vocabulary as an outcome. These 14 papers will be further screened at stage 2 of the screening process (full text stage, outlined below).

The original review search strategy (29) has been updated for the current review, accounting for advances in terminology, e.g., consensus on the term 'Developmental Language Disorder' (2). The original 'Child Talk' search encompassed a broader range of speech and language outcomes, therefore the search terms for the current review have been adjusted to focus on our two specific outcomes of interest; oral vocabulary and speech comprehensibility. The updated search strategy was initially reviewed by two independent post-doctoral researchers in the field and adjusted as needed, for example, adding in the term 'specific language impairment', which may be relevant to older papers in the search. For the revised search strategy draft for each database, please see supplementary material 1.

Study records, selection and data collection process

Search results will initially be imported into RefWorks, where duplicates will be removed by the first author (LR). The remaining articles will then be uploaded to the Covidence systematic review management database.

Initially, the first author (LR) will trial the exclusion guidance criteria document on 30 papers. For the initial draft of this exclusion criteria guidance document, please see supplementary material 2. The 30 papers will be randomly selected using a random number generator. These 30 papers will then be reviewed by a second reviewer. The reviewers will then meet to discuss discrepancies and make amendments to the exclusion guidance document if needed.

For peer review only

The screening and data extraction will be carried out as follows:

1) *Title/abstract screening*
The full set of titles/abstracts will be screened by the first author (LR). If uncertainty arises about how to apply to eligibility criteria to a specific paper, these articles will be discussed with a member of the review team (who is not involved in the formal screening process). This may then lead to further revisions to the exclusion criteria document. Following this, a second independent reviewer (SH) will independently screen the titles/abstracts. Any disagreements, and how these may relate to the exclusion guidance document, will be discussed in consensus meetings. Any disputed articles will then be re-screened should alterations have been made to the exclusion criteria document. If disagreement is not caused by confusion over the exclusion criteria document, and consensus cannot be reached through discussion, a third reviewer will be consulted.

2) *Full paper screening*
At the full text screening stage, two reviewers (LR, SH) will independently appraise all of the remaining articles for inclusion, following the iterative process as outlined for stage 1.

To enable transparency of the reliability of screening at stages 1 and 2, Cohen's K for these stages will be reported in the final paper.

3) *Risk of bias/internal validity*
Retained studies will then undergo assessment of internal validity by two independent reviewers (LR, SH). The reviewers will have regular consensus meetings, after independently assessing up to 4 papers at a time, to resolve potential conflicts. If disagreements persist, a third reviewer will be involved. Disagreements that arise (including those that have been resolved) will be recorded and reported in the final paper.

For the PEDro-P (43), papers with a rating of six and over will be retained for data extraction. This aligns with the original review (29). On the Risk of Bias in N-of-1 Trials (RoBiNT) scale, included studies will be rated as *fair* or above (44).

4) *Data extraction*
The first author (LR) will extract data from the first 25% of studies. These will be randomly selected using random number generation. A second extractor (SH) will then independently extract data from the same studies. The two extractors will then meet to discuss potential discrepancies, and to update the data extraction form if needed. Following this, the first author (LR) will extract the remainder of the data.

Data items
Data will be sought regarding general study information (e.g., date; study type; location; participant numbers), population characteristics (e.g., male/female; age; speech/language profile-including phonological SSD subtype), intervention techniques (e.g., dosage;

For peer review only

underpinning theory and justification given by the authors) and reported impact on the outcome of interest. Data on reported participant SES background will also be obtained, due to this being a known risk factor within developmental speech and language disorders (45). We will also collate information on the number of languages spoken by the participants, as well as reported ethnicities, with this being a potential factor for the external validity of findings (i.e., relevance to 'everyday' clinical practice).

The Template for Intervention Description and Replication (TIDieR) will be used as a framework to guide the extraction process (46), combined with the speech and language therapy specific 'Dose form Framework and Definitions', based on the work of Warren and colleagues (47); this has been applied in other reviews specific to paediatric speech and language therapy intervention (41). Details of techniques will be extracted regarding intervention contexts (e.g. the overarching activity the technique is presented in), method of instruction (e.g. who delivers the technique, where and when), and technique dosage (dose frequency and dose duration). All reported dosage information will be extracted in order to allow for variation in study design; most notably, studies which target both oral vocabulary and speech comprehensibility concurrently.

Outcomes and prioritisation
The two outcomes (oral vocabulary, speech comprehensibility) are of equal interest within this review, regardless of whether they are primary or secondary outcomes within the included studies.

Risk of bias in individual studies
Individual studies will be assessed for internal validity. To encompass the range of study designs included within this review, we will use the PEDro-P (43). Specifically, for single case experimental designs, the RoBiNT scale will be used (44).

Data synthesis
Quantitative data
Overarching details for each included study, including the individual internal validity ratings, will be given in the first table. Two summary graphs will also be presented to convey the percentage of overall ratings from the PEDro-P and RoBiNT Scales. The frequency of techniques within the included papers will be presented either numerically within a table, or within a graph or chart if this deemed more suited to the data collected. We will be guided by the synthesis without meta-analysis (SWiM) in systematic review guidelines (48) and will report on the direction of effect of the interventions, using vote counting with a sign test if appropriate.

Qualitative data
A description of the identified techniques will be presented in a table, including details regarding how they were operationalised, based on TIDieR (46) and the dose form framework (30,47).

The narrative synthesis will include sections on:
1)Similarities and differences (including shared core characteristics) between techniques used for the different outcomes

2)Patterns of technique dosage and delivery across the interventions
3)How the similarities and differences (including shared core characteristics) in techniques relate to underlying theory. Depending on findings, this section will be broken down into sub sections focusing on each theory of interest, potentially including (but not necessarily limited to):
- the lexical restructuring hypothesis
- the speech processing model
- the neural basis for speech and language development
4)The effectiveness of interventions which contain these techniques/shared core characteristics of techniques

If relevant, any observed differences between interventions for different phonological SSD sub types will be incorporated into the narrative synthesis, or given in an additional section if deemed to be more appropriate to the data found.

In the event of lab-based training studies meeting the final inclusion criteria, this data will be presented on a separate table. Additionally, within the narrative synthesis itself they will not be directly compared to the effectiveness studies. Instead, they will be used to support any potential theory building arising from the synthesis.

If challenges are identified regarding gaps and quality in the knowledge base, this will also be explored within the results and discussion section of the corresponding results paper.

Patient and public involvement
According to the James Lind Alliance, knowing how to best select communication strategies according to a child's individual profile is the 2nd most important recommendation for research (49). This is strongly in keeping with the aims of this review, and highlights the broader relevance of this work.

For our wider intervention development study, outcomes were prioritised by clinicians and parents of pre-school children with DLD/SSD within pre-study PPIE work (50,51). They identified the outcomes of increasing 1) oral vocabulary *(DLD outcome)*, and 2) speech comprehensibility *(SSD outcome)*. This provides further support focusing on techniques that directly target oral vocabulary and speech comprehensibility.

In keeping with the integral role of PPIE throughout, a newly formed project PPIE steering group will provide input at key points in the review process. This is a diverse group consisting of parents, speech and language therapists, a person with DLD, a specialist early years educator, a bi/multi-lingual educational family support worker and a clinical equality, diversity and inclusion (EDI) expert. During the review, they will be involved with:

1) Reviewing the content for the data extraction form (supplementary document 3), prior to the data extraction phase

2) Identifying what data has the most relevance in the 'real world', with these potentially informing recommendations within the final paper

For peer review only - http://bmjopen.bmj.com/site/about/guidelines.xhtml

3) Defining and agreeing key messages to take from the review, and dissemination through the steering group networks.

Steering group input will be recorded and reported in the final article, in accordance with the GRIPP 2 reporting criteria short form (52).

Meta-bias(es)
It is important to acknowledge that meta-bias, including reporting and publication bias, is present within all aspects of health research. Although it is not possible to completely control for such bias, we will:

1) Establish if the protocol for each study was published before recruitment for participants commenced (where possible)

2) Compare the outcomes and results sections of the published report when a protocol is available (for when considering selective reporting bias)

3) Assess potential publication bias through the inclusion of prioritised grey literature (thesis/dissertations)

Confidence in cumulative evidence
Confidence within the evidence as a whole will be based on the summary of the internal validity, as presented in the two summary tables (see data synthesis section). We will also acknowledge and discuss key factors relating to meta bias, and how the review findings should be interpreted based on this.

ETHICS AND DISSEMINATION
As a systematic review this study does not warrant ethics board approval. Findings will be disseminated though peer reviewed publications, social media, and project steering group networks.

*** ***

Contributors
LR led on this work and independently developed an initial draft of the manuscript (and appended documents). RH, NB, MC and SH suggested amendments after reviewing this and 3 subsequent re-drafts by LR. LR completed the fourth and final version of the manuscript, which was reviewed and agreed by all of the authors. LR led on manuscript revisions following peer review and all authors agreed to the final submitted version.

Acknowledgements
The authors would also like to acknowledge and thank Dr Pauline Frizelle (University College Cork) and Dr Helen Stringer (Newcastle University) for providing expert independent reviews of the initial terms for the search strategy.

Competing interests
There are no competing interests to declare.

For peer review only

Funding

This research was funded in whole, or in part, by the Wellcome Trust (Grant number 223500/Z/21/Z). For the purpose of open access, the author has applied a CC BY public copyright licence to any Author Accepted manuscript version arising from this submission.

REFERENCES

1)Norbury, C.F., Gooch, D., Wray, C., Baird, G., Charman, T., Simonoff, E., Vamvakas, G., Pickles, A., 2016. The impact of nonverbal ability on prevalence and clinical presentation of language disorder: Evidence from a population study. https://doi.org/10.1111/jcpp.12573

2)Bishop, D.V., Snowling, M.J., Thompson, P.A., Greenhalgh, T., Catalise-2 Consortium, Adams, C., Archibald, L., Baird, G., Bauer, A., Bellair, J., Boyle, C., 2017. Phase 2 of CATALISE: A multinational and multidisciplinary Delphi consensus study of problems with language development: Terminology. https://doi.org/10.1111/jcpp.12721

3)Duff, F.J., Reen, G., Plunkett, K., Nation, K., 2015. Do infant vocabulary skills predict school-age language and literacy outcomes?. Journal of Child Psychology and Psychiatry, 56(8), pp.848-856. https://doi.org/10.1111/jcpp.12378

4)Manning BL, Roberts MY, Estabrook R, Petitclerc A, Burns JL, Briggs-Gowan M, Wakschlag LS, Norton ES., 2019. Relations between toddler expressive language and temper tantrums in a community sample. Journal of Applied Developmental Psychology, volume 65, article 101070. https://doi.org/10.1016/j.appdev.2019.101070

5)Dodd, B., Reilly, S., Ttofari Eecen, K., Morgan, A.T., 2018. Articulation or phonology? Evidence from longitudinal error data. https://doi.org/10.1080/02699206.2018.1488994

6)Eadie, P., Morgan, A., Ukoumunne, O.C., Ttofari Eecen, K., Wake, M., Reilly, S., 2015. Speech sound disorder at 4 years: Prevalence, comorbidities, and predictors in a community cohort of children. https://doi.org/10.1111/dmcn.12635

7)McCormack J, McLeod S, McAllister L, Harrison LJ. My speech problem, your listening problem, and my frustration: The experience of living with childhood speech impairment. Speech, Language and hearing Services in Schools, *41*(4), pp.379-392. https://doi.org/10.1044/0161-1461(2009/08-0129)

8)Pommée, T., Balaguer, M., Mauclair, J., Pinquier, J., Woisard, V., 2022. Intelligibility and comprehensibility: A Delphi consensus study. https://doi.org/10.1111/1460-6984.12672

9)To, C.K.S., McLeod, S., Sam, K.L., Law, T., 2022. Predicting Which Children Will Normalize Without Intervention for Speech Sound Disorders. *Journal of Speech, Language, and Hearing Research*, *65*(5), pp.1724-1741. https://doi.org/10.1044/2022_JSLHR-21-00444

For peer review only - http://bmjopen.bmj.com/site/about/guidelines.xhtml

10) Burgoyne K, Lervag A, Malone S, Hulme C. 2019. Speech difficulties at school entry are a significant risk factor for later reading difficulties. Early Childhood Research Quarterly. *1* (49), pp. 40-8. https://doi.org/10.1016/j.ecresq.2019.06.005

11)Jin F, Schjølberg S, Wang MV, Eadie P, Nes RB, Røysamb E, Tambs K., 2020. Predicting literacy skills at 8 years from preschool language trajectories: A population-based cohort study. Journal of Speech, Language, and Hearing Research,*63(*8), pp. 2752-62. https://doi.org/10.1044/2020_JSLHR-19-00286

12)Hustad KC, Mahr TJ, Natzke P, Rathouz PJ. 2021. Speech development between 30 and 119 months in typical children I: Intelligibility growth curves for single-word and multiword productions. Journal of Speech, Language, and Hearing Research, *64*(10), pp.3707-19. https://doi.org/10.1044/2021_JSLHR-21-00142

13)Shriberg, L.D., Kwiatkowski, J., 1994. Developmental phonological disorders I: A clinical profile. https://doi.org/10.1044/jshr.3705.1100

14)Broomfield, J., Dodd, B., 2004. Children with speech and language disability: caseload characteristics. https://doi.org/10.1080/13682820310001625589

15)Hayiou-Thomas, M.E., Carroll, J.M., Leavett, R., Hulme, C., Snowling, M.J., 2017. When does speech sound disorder matter for literacy? The role of disordered speech errors, co-occurring language impairment and family risk of dyslexia. https://doi.org/10.1111/jcpp.12648

16)Lewis, B.A., Freebairn, L., Tag, J., Ciesla, A.A., Iyengar, S.K., Stein, C.M., Taylor, H.G., 2015. Adolescent outcomes of children with early speech sound disorders with and without language impairment. https://doi.org/10.1044/2014_AJSLP-14-0075

17)Wren, Y., Miller, L.L., Peters, T.J., Emond, A., Roulstone, S., 2016. Prevalence and predictors of persistent speech sound disorder at eight years old: Findings from a population cohort study. https://doi.org/10.1044/2015_JSLHR-S-14-0282

18)Johnson, C.J., Beitchman, J.H., Brownlie, E.B., 2010. Twenty-year follow-up of children with and without speech-language impairments: Family, educational, occupational, and quality of life outcomes. https://doi.org/10.1044/1058-0360(2009/08-0083)

19)Le, H.N., Mensah, F., Eadie, P., McKean, C., Sciberras, E., Bavin, E.L., Reilly, S., Gold, L., 2021. Health-related quality of life of children with low language from early childhood to adolescence: Results from an Australian longitudinal population-based study. https://doi.org/10.1111/jcpp.13277

20)St Clair, M.C., Forrest, C.L., Yew, S.G.K., Gibson, J.L., 2019. Early risk factors and emotional difficulties in children at risk of developmental language disorder: A population cohort study. https://doi.org/10.1044/2018_JSLHR-L-18-0061

21)Dodd, B., 2014. Differential diagnosis of pediatric speech sound disorder. https://doi.org/10.1007/s40474-014-0017-3

22)Petinou, K., Taxitari, L., Phinikettos, I., Theodorou, E., 2021. Dynamic linguistic interconnectedness and variability in toddlers. https://doi.org/10.1007/s10936-020-09747-y

23)Stoel-Gammon, C., 2011. Relationships between lexical and phonological development in young children. https://doi.org/10.1017/S0305000910000425

24)Ainsworth, S., Welbourne, S., Hesketh, A., 2016. Lexical restructuring in preliterate children: Evidence from novel measures of phonological representation. https://doi.org/10.1017/S0142716415000338

25)Pascoe, M., Stackhouse, J., Wells, B. Persisting Speech Difficulties in Children: Children's Speech and Literacy Difficulties. Chichester, UK: Whurr Publishers limited 2006: page 383

26)Rodgers, L., Harding, S., Rees, R., Clarke, M.T., 2022. Interventions for pre-school children with co-occurring phonological speech sound disorder and expressive language difficulties: A scoping review. https://doi.org/10.1111/1460-6984.12719

27)Tyler AA, Lewis KE, Haskill A, Tolbert LC. Efficacy and cross-domain effects of a morphosyntax and a phonology intervention. 2002. Language, Speech, and Hearing Services in Schools. https://doi.org/10.1044/0161-1461(2002/005)

28)Department for Education. 2012. Exploring interventions for children and young people with speech, language and communication needs: A study of practice. https://assets.publishing.service.gov.uk/government/uploads/system/uploads/attachment_data/file/219627/DFE-RR247-BCRP13.pdf

29)Roulstone, S.E., Marshall, J.E., Powell, G.G., Goldbart, J., Wren, Y.E., Coad, J., Daykin, N., Powell, J.E., Lascelles, L., Hollingworth, W., Emond, A., 2015. Evidence-based intervention for preschool children with primary speech and language impairments: Child Talk–an exploratory mixed-methods study. https://doi.org/10.3310/pgfar03050

30)Frizelle, P., McKean, C., 2022. Using Theory to Drive Intervention Efficacy: The Role of Dose Form in Interventions for Children with DLD. https://doi.org/10.3390/children9060859

31)Morgan, L., Marshall, J., Harding, S., Powell, G., Wren, Y., Coad, J., Roulstone, S., 2019. 'It depends': Characterizing speech and language therapy for preschool children with developmental speech and language disorders. https://doi.org/10.1111/1460-6984.12498

32)Girolametto L, Pearce PS, Weitzman E. 1995. The effects of focused stimulation for promoting vocabulary in young children with delays: A pilot study. Journal of Children's Communication Development, *17*(2), pp.39-49. https://doi.org/10.1177/152574019501700205

33)Romeo, R.R., Leonard, J.A., Robinson, S.T., West, M.R., Mackey, A.P., Rowe, M.L., Gabrieli, J.D., 2018. Beyond the 30-million-word gap: Children's conversational exposure is associated with language-related brain function. https://doi.org/10.1177/0956797617742725

34)Wasik, B. A., Jacobi-Vessels, J. L. (2017). Word play: Scaffolding language development through child-directed play. *Early Childhood Education Journal, 45*(6), pp. 769-776. https://doi.org/10.1007/s10643-016-0827-5

35)Zimmerman, I. L., Steiner, V. G., Pond, R. E. (2002). *Preschool Language Scale, Fourth Edition (PLS-4)*.https://doi.org/10.1037/t15140-000

36)McLeod, S., Harrison, L.J., McCormack, J., 2012. The intelligibility in context scale: Validity and reliability of a subjective rating measure. https://doi.org/10.1044/1092-4388(2011/10-0130)

37)Curran, M., Owen Van Horne, A., 2019. Use of Recast Intervention to Teach Causal Adverbials to Young Children With Developmental Language Disorder Within a Science Curriculum: A Single Case Design Study. https://doi.org/10.1044/2018_AJSLP-17-0164

38)Sugden, E., Baker, E., Williams, A.L., Munro, N., Trivette, C.M., 2020. Evaluation of parent-and speech-language pathologist–delivered multiple oppositions intervention for children with phonological impairment: A multiple-baseline design study. https://doi.org/10.1044/2019_AJSLP-18-0248

39)Yoder, P.J., Woynaroski, T., Camarata, S., 2016. Measuring speech comprehensibility in students with Down syndrome. *Journal of Speech, Language, and Hearing Research*, *59*(3), pp.460-467. https://doi.org/10.1044/2015_JSLHR-S-15-0149

40)Wren, Y., Harding, S., Goldbart, J., Roulstone, S., 2018. A systematic review and classification of interventions for speech-sound disorder in preschool children. https://doi.org/10.1111/1460-6984.12371

41)Frizelle, P., Tolonen, A.K., Tulip, J., Murphy, C.A., Saldana, D., McKean, C., 2021. The influence of quantitative intervention dosage on oral language outcomes for children with developmental language disorder: A systematic review and narrative synthesis. https://doi.org/10.1044/2020_LSHSS-20-00058

42)Carson, L., Baker, E. and Munro, N., 2022. A Systematic Review of Interventions for Late Talkers: Intervention Approaches, Elements, and Vocabulary Outcomes. https://doi.org/10.1044/2022_AJSLP-21-00168

43)Murray, E., Power, E., Togher, L., McCabe, P., Munro, N., Smith, K., 2013. The reliability of methodological ratings for speechBITE using the PEDro-P scale. https://doi.org/10.1111/1460-6984.12007

44)Tate, R.L., Perdices, M., Rosenkoetter, U., Wakim, D., Godbee, K., Togher, L., McDonald, S., 2013. Revision of a method quality rating scale for single-case experimental designs and n-of-1 trials: The 15-item Risk of Bias in N-of-1 Trials (RoBiNT) Scale. https://doi.org/10.1080/09602011.2013.824383

45)Law, J., Charlton, J., Dockrell, J., Gascoigne, M., McKean, C., Theakston, A., 2017. Early Language Development: Needs, provision and intervention for pre-school children from socio-economically disadvantaged backgrounds. London: Education Endowment Foundation. https://educationendowmentfoundation.org.uk/public/files/Law_et_al_Early_Language_Development_final.pdf

46)Hoffmann, T.C., Glasziou, P.P., Boutron, I., Milne, R., Perera, R., Moher, D., Altman, D.G., Barbour, V., Macdonald, H., Johnston, M., Lamb, S.E., 2014. Better reporting of interventions: template for intervention description and replication (TIDieR) checklist and guide. https://doi.org/10.1136/bmj.g1687

47)Warren, S.F., Fey, M.E., Yoder, P.J. 2007. Differential treatment intensity research: A missing link to creating optimally effective communication interventions. https://doi.org/10.1002/mrdd.20139

48)Campbell, M., McKenzie, J.E., Sowden, A., Katikireddi, S.V., Brennan, S.E., Ellis, S., Hartmann-Boyce, J., Ryan, R., Shepperd, S., Thomas, J., Welch, V., 2020. Synthesis without meta-analysis (SWiM) in systematic reviews: reporting guideline. https://doi.org/10.1136/bmj.l6890

49)Morris C, Simkiss D, Busk M*, et al.*Setting research priorities to improve the health of children and young people with neurodisability: a British Academy of Childhood Disability-James Lind Alliance Research Priority Setting Partnership. doi: 10.1136/bmjopen-2014-006233

50)Rodgers, L., and Harford., D. Collaborating with parents to identify outcomes for a preschool speech and language intervention. ESLA Congress 2022: New frontiers in speech and language therapy: advancing practice, research and education. DOI:10.13140/RG.2.2.34626.45763 https://www.researchgate.net/publication/362910019_Collaborating_with_parents_to_identify_outcomes_for_a_pre-school_speech_and_language_intervention

51)Rodgers, L., and Harford., D. 2022. Part of the family. RCSLT Bulletin. Issue 832, autumn 2022.

52)Staniszewska, S., Brett, J., Simera, I., Seers, K., Mockford, C., Goodlad, S., Altman, D.G., Moher, D., Barber, R., Denegri, S. and Entwistle, A., 2017. GRIPP2 reporting checklists: tools to improve reporting of patient and public involvement in research. https://doi.org/10.1136/bmj.j3453

FIGURE TITLE

Figure 1. Speech, language and communication needs (2)

For peer review only

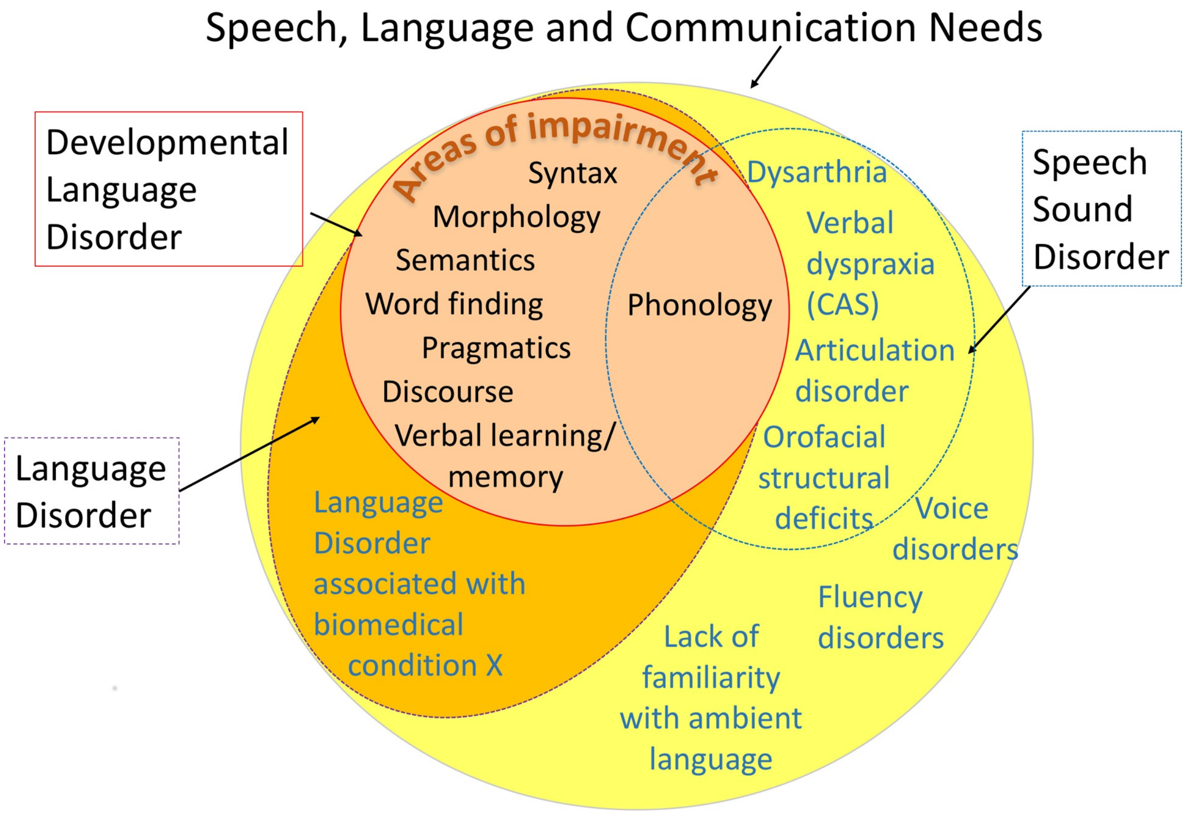
Speech, Language and Communication Needs
Developmental Language Disorder
Areas of impairment
Syntax
Morphology
Semantics
Word finding
Pragmatics
Discourse
Verbal learning/ memory
Phonology
Dysarthria
Verbal dyspraxia (CAS)
Articulation disorder
Orofacial structural deficits
Voice disorders
Fluency disorders
Speech Sound Disorder
Language Disorder
Language Disorder associated with biomedical condition X
Lack of familiarity with ambient language

SUPPLEMENTARY MATERIALS 1:
SEARCH STRATEGY FOR EACH DATABASE

1.EBSCO *(Medline, APA Psychinfo, CINAHL, Communication Source)*

Paediatric **OR** paediatrics **OR** children **OR** child **OR** infant **OR** infants **OR** schoolchild **OR** schoolchildren **OR** preschool **OR** “early years” **OR** kindergarten *(AB)*
NOT
Teenage **OR** teenagers **OR** adolescent **OR** adolescents *(SU)*

AND

Therapy **OR** intervention **OR** interventions **OR** treatment **OR** treatments **OR** programme **OR** programmes **OR** program **OR** programs **OR** teaching **OR** instruction **OR** approach **OR** approaches **OR** technique **OR** techniques **OR** strategy **OR** strategies **OR** activity **OR** activities **OR** class **OR** classes *(AB)*

AND

“language delay” **OR** “language disorder” **OR** “specific language impairment” **OR** “language impairment” **OR** “language difficulties” **OR** “developmental language disorder” **OR** “late talker” **OR** “speech delay” **OR** “speech disorder” **OR** “speech sound disorder” **OR** “speech intelligibility” **OR** “intelligible speech” **OR** “speech comprehensibility” **OR** “comprehensible speech” *(AB)*
NOT
“sign language” **OR** “mental retardation” **OR** autism **OR** “autistic spectrum disorder” **OR** Asperger **OR** “cleft lip” **OR** “cleft palate” **OR** deaf **OR** “cerebral palsy” **OR** aphonia **OR** geriatrics **OR** “down syndrome” **OR** “cochlear implant” **OR** “autistic spectrum disorder” **OR** “autistic spectrum disorders” **OR** “autism disorder” **OR** “autistic disorder” (*SU)*

Limiters: 2012-current

2.Ovid *(Emcare)*
Paediatric **OR** paediatrics **OR** children **OR** child **OR** infant **OR** infants **OR** schoolchild **OR** schoolchildren **OR** preschool **OR** “early years” **OR** kindergarten *(AB)*
NOT
Teenage **OR** teenagers **OR** adolescent **OR** adolescents *(SH)*

AND

Therapy **OR** intervention **OR** interventions **OR** treatment **OR** treatments **OR** programme **OR** programmes **OR** program **OR** programs **OR** teaching **OR** instruction **OR** approach **OR** approaches **OR** technique **OR** techniques **OR** strategy **OR** strategies **OR** activity **OR** activities **OR** class **OR** classes *(AB)*

For peer review only

AND

“language delay” **OR** “language disorder” **OR** “specific language impairment” **OR** “language impairment” **OR** “language difficulties” **OR** “developmental language disorder” **OR** “late talker” **OR** “speech delay” **OR** “speech disorder” **OR** “speech sound disorder” **OR** “speech intelligibility” **OR** “intelligible speech” **OR** “speech comprehensibility” **OR** “comprehensible speech” *(AB)*
NOT
“sign language” **OR** “mental retardation” **OR** autism **OR** “autistic spectrum disorder” **OR** Asperger **OR** “cleft lip” **OR** “cleft palate” **OR** deaf **OR** “cerebral palsy” **OR** aphonia **OR** geriatrics **OR** “down syndrome” **OR** “cochlear implant” **OR** “autistic spectrum disorder” **OR** “autistic spectrum disorders” **OR** “autism disorder” **OR** “autistic disorder” (*SH*)

Limiters: 2012-current

3.ERIC (descriptor terms)

"speech impairments" **OR** "language impairments" **OR** intelligibility **OR** "expressive language" **AND** "speech language pathology" **OR** "speech therapy" **AND** "young child" **OR** "preschool children" **OR** toddlers **OR** "early childhood education”

Limiters: peer reviewed only
Pre-2012 studies to be removed manually following the search, within Refworks

For peer review only

SUPPLEMENTARY MATERIALS 2: EXCLUSION SCREENING GUIDANCE

Population

Inclusion

80% children aged 2:0 and 5:11; idiopathic phonological speech production difficulties and/or difficulties relating to oral vocabulary; difficulties identified on standardised assessments, parental/and or professional observation reports, and/or pre-intervention baseline probes; idiopathic speech/language needs

Exclusion

Children with typically developing speech/language skills; speech/language difficulties not caused by or associated with a condition with a known impact on communication e.g. autism, deafness, cerebral palsy, cleft lip/palate, dysarthria

Title/abstract level specific: still include if any of the exclusion criteria remains unknown and the inclusion criteria is met or unknown.

Intervention

Inclusion: Any setting, deliverer, mode of delivery

Exclusion: N/A

Comparator

Inclusion: Empirical evaluation of intervention effectiveness from RCTs, experimental and quasi-experimental studies, case studies/within groups designs.

Exclusion: Assessment at a single timepoint pre and post intervention, with no comparator.

Title/abstract level specific: still include if any of the exclusion criteria remains unknown and the inclusion criteria is met or unknown.

Outcome

Inclusion: Measure of oral vocabulary and/or speech comprehensibility. Include intelligibility measures as a proxy for comprehensibility. Include proximal measures of vocabulary development that may arise from syntactic assessments, such as the number of different words (NDW). Outcomes must be evaluated via standardised assessment, probes, and/or observational ratings or scales. Post intervention assessment at any timepoint.

Exclusion: Composite measures where individual results for speech comprehensibility/intelligibility and/or oral vocabulary are not completed in a separate analysis. Purely syntactic measures of change, such as the mean length of utterance in morphemes (MLUm).

Title/abstract level specific: still include if any of the exclusion criteria remains unknown and the inclusion criteria is met or unknown. Still include if primary outcomes do not relate to

For peer review only

vocabulary/speech comprehensibility, but where the inclusion criteria are either met or unknown.

For peer review only

SUPPLEMENTARY MATERIALS 3:
DRAFT CONTENT FOR THE DATA EXTRACTION FORM

1.General information/study details

- Date
- Title
- Location (country)
- Language the intervention was in
- Study design
- Comparator
- No. participants (incl. Participants in control/alternative experimental group if relevant)
- Why- goals/aims of the overall intervention
- Specific outcome/s measured of relevance to this review
- Is this a primary outcome of the intervention?
- Do the authors refer to a protocol which was made available before recruitment commenced?
- If a pre-study protocol exists, do the outcomes and results section of the published report align with this protocol?

2.Population characteristics

- Age
- Male/female
- Languages spoken
- Ethnicity
- SES details as reported (e.g. parental education/employment)
- Assessments used to identify DLD/SSD features
- Pre-intervention speech assessment levels
- Pre-intervention language assessment levels (comprehension as well as expressive)
- Phonological SSD sub type- was this specified?
- If phonological SSD sub type was not specified, was it indicated-and if so, how? *(e.g. through the selection of treatment targets, baseline assessment results)*

3.Intervention characteristics

- Setting (e.g. home, nursery/school, clinic)
- Was the intervention modified at any point? If so, how?
- Techniques within the intervention
- Rationale for each technique/the technique within the wider approach
- Mode of delivery of the technique (e.g. fully face to face in clinic; hybrid in clinic with some carryover over home and/or nursery; virtual delivery)
- Was technique delivery implicit (e.g. listening to an adults' model) or explicit (e.g. being asked to repeat)?
- The wider activity/game the technique is part of (e.g. shared book reading, child led play, everyday routines, a combination of these)

For peer review only

- Dose frequency of the individual technique (no. times delivered per session, day, across a week)
- Dose frequency of the intervention as a whole
- Total duration of the technique (the time period between which the technique is used)
- Total duration of the intervention (the time period/duration of the intervention as a whole)
- Additional dosage information as reported
- Deliverer/s of the technique
- If not SLT, how the person was trained to deliver the technique
- Materials used to carry out the technique
- Was intervention fidelity measured? Report on this if so

4.Outcomes

- Summary of exact outcome *(e.g. comprehensibility with parents or with teaching staff? Spontaneous word production in free play or picture naming?)*
- Measure/s used
- Timepoints
- Reported effect
- Direction of effect (benefit vs no benefit/harm)

For peer review only

PRISMA-P (Preferred Reporting Items for Systematic review and Meta-Analysis Protocols) 2015 checklist: recommended items to address in a systematic review protocol*

Section and topic	Item No	Checklist item	Location
**ADMINISTRATIVE INFORMATION**			
Title:			
Identification	1a	Identify the report as a protocol of a systematic review	Page 1 (title page)
Update	1b	If the protocol is for an update of a previous systematic review, identify as such	Page 2 (abstract) further details on page 5 (broader context: an intervention development study)
Registration	2	If registered, provide the name of the registry (such as PROSPERO) and registration number	Page 2 (abstract), page 6
Authors:			
Contact	3a	Provide name, institutional affiliation, e-mail address of all protocol authors; provide physical mailing address of corresponding author	Page 1 (title page)
Contributions	3b	Describe contributions of protocol authors and identify the guarantor of the review	Pages 13/14
Amendments	4	If the protocol represents an amendment of a previously completed or published protocol, identify as such and list changes; otherwise, state plan for documenting important protocol amendments	Page 2 (abstract); further details on page 9 (search strategy)
Support:			
Sources	5a	Indicate sources of financial or other support for the review	Page 14
Sponsor	5b	Provide name for the review funder and/or sponsor	Page 14
Role of sponsor or funder	5c	Describe roles of funder(s), sponsor(s), and/or institution(s), if any, in developing the protocol	Page 14
**INTRODUCTION**			
Rationale	6	Describe the rationale for the review in the context of what is already known	Pages 3-6 (introduction)
Objectives	7	Provide an explicit statement of the question(s) the review will address with reference to participants, interventions, comparators, and outcomes (PICO)	Page 6 (objectives and research questions) further details on pages 7/8 (eligibility criteria)
**METHODS**			
Eligibility criteria	8	Specify the study characteristics (such as PICO, study design, setting, time frame) and report characteristics (such as years considered, language, publication status) to be used as criteria for eligibility for the review	Page 6 (objectives and research questions); further details on

For peer review only

			pages 7/8 (eligibility criteria)
Information sources	9	Describe all intended information sources (such as electronic databases, contact with study authors, trial registers or other grey literature sources) with planned dates of coverage	Pages 8-9 (information sources)
Search strategy	10	Present draft of search strategy to be used for at least one electronic database, including planned limits, such that it could be repeated	Supplementary materials document 1
Study records:			
Data management	11a	Describe the mechanism(s) that will be used to manage records and data throughout the review	Pages 9/10 (study records, selection and data collection process)
Selection process	11b	State the process that will be used for selecting studies (such as two independent reviewers) through each phase of the review (that is, screening, eligibility and inclusion in meta-analysis)	Pages 9/10 (study records, selection and data collection process)
Data collection process	11c	Describe planned method of extracting data from reports (such as piloting forms, done independently, in duplicate), any processes for obtaining and confirming data from investigators	Page 10 (study records, selection and data collection process); page 11 (data items); supplementary materials document 3
Data items	12	List and define all variables for which data will be sought (such as PICO items, funding sources), any pre-planned data assumptions and simplifications	Page 11(data items) supplementary materials document 3
Outcomes and prioritization	13	List and define all outcomes for which data will be sought, including prioritization of main and additional outcomes, with rationale	Page 11 (outcomes and prioritisation)
Risk of bias in individual studies	14	Describe anticipated methods for assessing risk of bias of individual studies, including whether this will be done at the outcome or study level, or both; state how this information will be used in data synthesis	Page 10 (risk of bias/internal validity); page 11 (risk of bias in individual studies); page 11 (quantitative data)
Data synthesis	15a	Describe criteria under which study data will be quantitatively synthesised	Page 11 (data synthesis: quantitative data)
	15b	If data are appropriate for quantitative synthesis, describe planned summary measures, methods of handling data and methods of combining data from studies, including any planned exploration of consistency (such as I^2, Kendall's τ)	Not applicable
	15c	Describe any proposed additional analyses (such as sensitivity or subgroup analyses, meta-regression)	Not applicable

For peer review only

	15d	If quantitative synthesis is not appropriate, describe the type of summary planned	Page 12 (data synthesis: qualitative data)
Meta-bias(es)	16	Specify any planned assessment of meta-bias(es) (such as publication bias across studies, selective reporting within studies)	Page 13 (meta-bias(es))
Confidence in cumulative evidence	17	Describe how the strength of the body of evidence will be assessed (such as GRADE)	Page 13 (strength in body of evidence)

*** It is strongly recommended that this checklist be read in conjunction with the PRISMA-P Explanation and Elaboration (cite when available) for important clarification on the items. Amendments to a review protocol should be tracked and dated. The copyright for PRISMA-P (including checklist) is held by the PRISMA-P Group and is distributed under a Creative Commons Attribution Licence 4.0.**

From: Shamseer L, Moher D, Clarke M, Ghersi D, Liberati A, Petticrew M, Shekelle P, Stewart L, PRISMA-P Group. Preferred reporting items for systematic review and meta-analysis protocols (PRISMA-P) 2015: elaboration and explanation. BMJ. 2015 Jan 2;349(jan02 1):g7647.

For peer review only
